# Mining a vibriophage depolymerase for enhanced pathogen control in aquaculture

**DOI:** 10.1128/aem.01824-25

**Published:** 2026-01-12

**Authors:** Yufei Yue, Jiulong Zhao, Zengmeng Wang, Rui Yin, Yang He, Chengcheng Li, Yongyu Zhang

**Affiliations:** 1 Qingdao New Energy Shandong Laboratory, Key Laboratory of Biofuels, Shandong Provincial Key Laboratory of Energy Genetics, Qingdao Institute of Bioenergy and Bioprocess Technology, Chinese Academy of Sciences 85424 , Qingdao, China; 2 University of Chinese Academy of Sciences 74519, Beijing, China; 3 Southern Marine Science and Engineering Guangdong Laboratory (Zhuhai) 590852 , Zhuhai, China; 4 Shandong Energy Institute, Qingdao, China; 5 Laboratory for Marine Biology and Biotechnology, Qingdao Marine Science and Technology Center 554912 , Qingdao, China; 6 Ocean University of China 12591, Qingdao, China; Indiana University Bloomington, Bloomington, Indiana, USA

**Keywords:** phage-derived depolymerase, Dep193, polysaccharide degradation, antibiofilm activity, phage synergy, *Vibrio *control

## Abstract

Despite the promise of phages as antibiotic alternatives, their efficacy is often undermined by the rapid emergence of bacterial resistance. Phage-derived enzymes, particularly depolymerases, offer a compelling strategy to overcome this limitation and enhance antibacterial therapy. Focusing on *Vibrio* pathogens, the major threats to global aquaculture, our bioinformatic analysis revealed that 79.4% of cultured and 46.2% of uncultured *Vibrio* phages encode putative depolymerases, underscoring a vast but underexploited antibacterial resource. We further isolated and characterized VnaP, a depolymerase-encoding phage (novel genus, *Caudovircetes*) that forms distinctive halo plaques indicative of depolymerase activity. Genome analysis identified ORF193, encoding a novel polysaccharide depolymerase lacking sequence or structural homology to any characterized depolymerases. Heterologously expressed Dep193 efficiently degraded *Vibrio* surface polysaccharides and exhibited potent antibiofilm activity. While Dep193 exhibits modest standalone antibacterial activity, its synergistic combination with VnaP significantly enhances bacterial clearance and delays resistance emergence across multiple *Vibrio* species. As the first biochemically validated *Vibrio* phage depolymerase, Dep193 broadens the known diversity of these enzymes and establishes an effective strategy for *Vibrio* control in aquaculture.

**IMPORTANCE:**

The rapid emergence of antibiotic-resistant *Vibrio* strains threatens global aquaculture sustainability, necessitating alternative antimicrobial strategies. This study identifies and characterizes Dep193, a novel phage-encoded depolymerase with polysaccharide-degrading and antibiofilm activities that enhances phage therapy efficacy through a previously unreported mechanism. The Dep193-phage VnaP combination exhibits broad-spectrum activity against multiple *Vibrio* species, demonstrating strong potential as a therapeutic strategy for aquaculture. Notably, Dep193 lacks any recognizable functional domains found in characterized depolymerases, representing the first validated member of a novel evolutionary clade. These findings expand the known diversity of phage depolymerases and provide a promising avenue for the targeted control of *Vibrio* infections in aquaculture.

## INTRODUCTION

The escalating global threat of antibiotic resistance has highlighted bacteriophages as promising alternatives for combating pathogenic bacteria. However, the rapid emergence of phage-resistant strains remains a major challenge (1, 2). In contrast to whole-phage treatment, phage-derived enzymes, such as endolysins, holins, and depolymerases, exert antibacterial effects with reduced propensity for inducing bacterial resistance, rendering them attractive candidates for antibacterial treatment (3–6).

Phage-encoded depolymerases are typically associated with phage tailspike or tail fiber proteins (7), or exist free (8–10). They target key bacterial surface polysaccharides, including capsular polysaccharides (CPS), lipopolysaccharides (LPS), and extracellular polysaccharides (EPS) (11). Such polysaccharides serve dual roles in bacterial pathogenicity: they function as major virulence factors and contribute to biofilm formation, collectively protecting bacteria from host immune defenses and antimicrobial agents (9, 12, 13). Based on their catalytic mechanisms, depolymerases are primarily categorized as either hydrolases, which cleave glycosidic bonds through hydrolysis, or lyases, which employ β-elimination reactions (6, 9, 14). By selectively disrupting these protective structures, depolymerases attenuate bacterial virulence without inducing extensive cell lysis (15), thereby minimizing the risk of endotoxin release and the associated inflammatory responses often triggered by lytic enzymes like endolysins and holins (16, 17). Beyond their anti-virulence properties, many depolymerases exhibit potent biofilm-degrading capabilities, addressing one of the most persistent challenges in bacterial infection control (14, 18). By disrupting the biofilm matrix, these enzymes enhance the penetration and effectiveness of conventional antimicrobials, facilitating more efficient bacterial clearance (11, 16, 19). Moreover, depolymerases can improve phage infectivity by degrading biofilm matrices that restrict viral access (20, 21) and by cleaving surface polysaccharides to expose secondary receptors that facilitate phage adsorption (14, 22). These multifaceted properties underscore the potential of depolymerases as next-generation antibacterial agents (7, 19).

*Vibrio* species are major pathogens in aquaculture, responsible for substantial economic losses and persistent management challenges (23–25). Polysaccharides on the surface of *Vibrio* cells have been identified as key virulence factors (26, 27), enabling the bacteria to withstand environmental stressors, antimicrobial compounds, and host immune responses (27–30). Depolymerases, found in 79.4% of cultured and 46.2% of uncultured *Vibrio* phages through our bioinformatic analysis, represent an important antibacterial enzyme resource but have rarely been explored. Here, we report the first biochemical validation of a *Vibrio* phage-derived depolymerase, Dep193, encoded by the novel lytic phage VnaP. Dep193 represents a previously unrecognized depolymerase family and exhibits dual functionality: it degrades host polysaccharides and synergizes with VnaP to enhance antibacterial efficacy and delay the emergence of resistance, directly addressing a major limitation of phage-based therapies. This study presents depolymerases as promising candidates for the development of novel strategies to control *Vibrio* infections in aquaculture.

## RESULTS

### High prevalence of depolymerases among *Vibrio* phages

Our analysis of public databases identified a total of 573 putative depolymerase genes encoded by *Vibrio* phages, comprising 215 from cultured RefSeq v210 database and 358 from uncultured IMG/VR v4 database (Table 1). Among the cultured *Vibrio* phages, at least one putative depolymerase gene was identified in 79.4% (81/102) of genomes. Notably, 55.8% (57/102) of these phages were predicted to encode two or more depolymerase candidates. Similarity analysis of uncultured *Vibrio* phages revealed that 46.2% (301/651) harbored at least one predicted depolymerase gene. This high prevalence of depolymerase across both cultured and uncultured *Vibrio* phages suggests that depolymerases represent a vast and largely untapped enzyme resource.

**TABLE 1:** Putative depolymerase gene prediction in *Vibrio* phages from RefSeq and IMG/VR databases

Database	Phage category	Total phages	Phages with ≥1 depolymerase	Total depolymerase genes
RefSeq	*Vibrio* phages	102	81 (79.4%)	215
IMG/VR	*Vibrio* phages	651	301 (46.2%)	358

### Characteristics of *Vibrio* phage VnaP with putative depolymerase activity

#### Morphological and lytic activity

**Fig 1:**
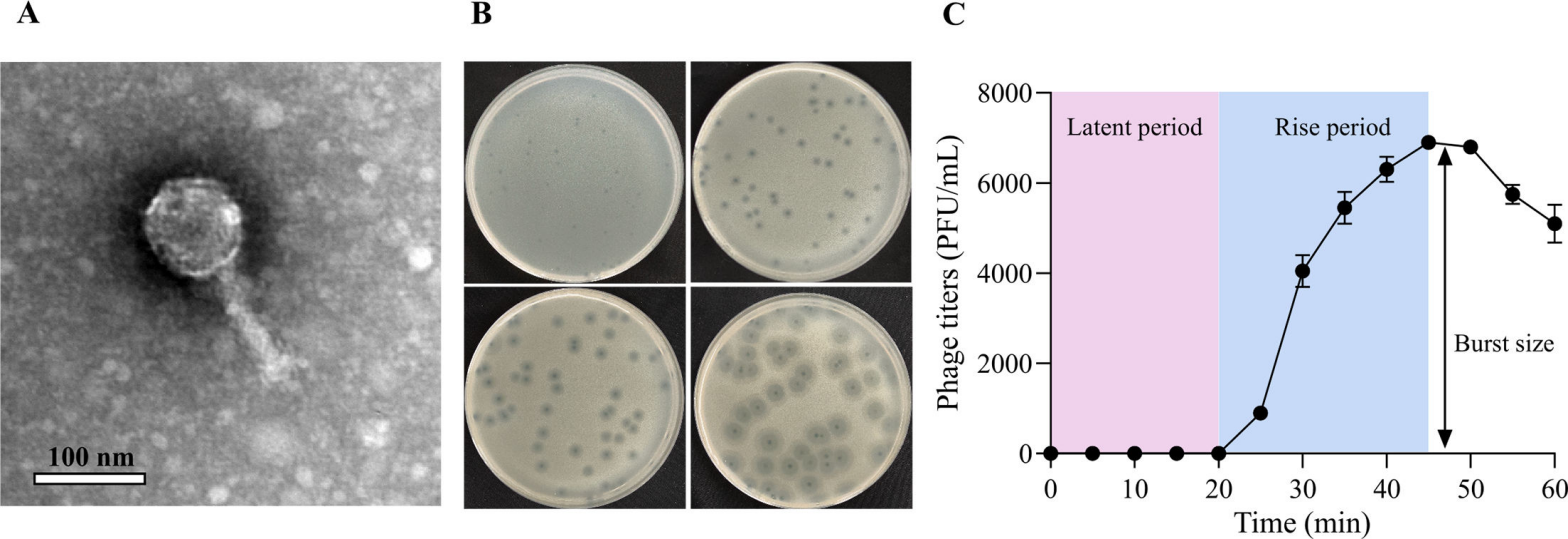
Biological characteristics of phage VnaP. (**A**) Transmission electron microscopy image of phage VnaP. (**B**) Temporal progression of plaque formation by phage VnaP on *V. natriegens* lawns. (**C**) One-step growth curve illustrating the infection dynamics of VnaP in *V. natriegens* AbY-1805. Data are shown as the mean ± SD (*n* = 3).

The phage VnaP was isolated from coastal waters of Qingdao, China, using *V. natriegens* AbY-1805 as host. Transmission electron microscopy (TEM) analysis revealed that VnaP possesses an icosahedral capsid (84 ± 2 nm in diameter) and a contractible tail (107 ± 2 nm in length) (Fig. 1A). Notably, VnaP formed plaques with a clear central lysis zone surrounded by fuzzy halos (Fig. 1B), a hallmark of depolymerases with moderate antibacterial activity that are released during phage lysis and readily diffuse beyond the primary infection zone due to their small size (31, 32). Resistance to chloroform treatment confirmed the absence of lipid-containing envelope in the virion structure (Fig. S1).

Host range characterization demonstrated that VnaP exhibits a broad lytic spectrum, effectively lysing *V. natriegens* AbY-1805, *V. diabolicus* YN5, *V. parahaemolyticus* WY7, *V. alginolyticus* SQ20, and *V. atypicus* BR18 (Table 2), highlighting its promising potential for application against multiple *Vibrio* pathogens. One-step growth curve (Fig. 1C) analysis revealed that VnaP had a short latent period (<20 min), followed by a rapid progeny release period (25 min), and produced a burst size of 110 PFU/cell.

**TABLE 2:** Host range of phage VnaP and bacterial specificity of depolymerase Dep193^*a*^

Strain	Most closely related strain	VnaP susceptibility	Dep193 activity
AbY-1805	*Vibrio natriegens*	+	+
YN5	*Vibrio diabolicus*	+	+
WY7	*Vibrio parahaemolyticus*	+	+
SQ20	*Vibrio alginolyticus*	+	+
BR18	*Vibrio atypicus*	+	+
ZZ006	*Vibrio sinaloensis*	−	−
VP	*Vibrio parahaemolyticus*	−	−
VIB645	*Vibrio harveii*	−	−
T-HJ001	*Vibrio scophthalmi*	−	−
HNX003	*Vibrio xuii*	−	−
FB1013	*Vibrio sagamiensis*	−	−
B1	*Vibrio owensii*	−	−
18-1	*Vibrio campbellii*	−	−
17S-2-3	*Vibrio hyugaensis*	−	−
283	*Vibrio alginolyticus*	−	−
YC18	*Vibrio aquaticus*	−	−
XT2	*Vibrio campbellii*	−	−
XT16	*Vibrio tubiashii*	−	−
XJK07	*Vibrio rumoiensis*	−	−
XJK009	*Vibrio rotiferianus*	−	−
XJK003	*Vibrio mytili*	−	−
X10S	*Vibrio neocaledonicus*	−	−
T-HJ003	*Vibrio renipiscarius*	−	−
PMP116	*Vibrio pacinii*	−	−
HS4-2	*Vibrio diabolicus*	−	−
HS031	*Vibrio azureus*	−	−
25013	*Vibrio variabilis*	−	−
XJK015	*Shewanella upenei*	−	−
XJK002	*Shewanella algae*	−	−
XFB1005	*Shewanella chilikensis*	−	−
X11S	*Acinetobacter johnsonii*	−	−
XT6	*Acinetobacter junii*	−	−
YC3N	*Acinetobacter haemolyticus*	−	−
XGQ	*Pseudomonas plecoglossicida*	−	−
ST203	*Tenacibaculum lutimaris*	−	−

^*a*^ +, plaque formation (for VnaP) or halo formation (for Dep193); −, no visible lytic or enzymatic activity.

### Genomic and phylogenetic analysis

**Fig 2:**
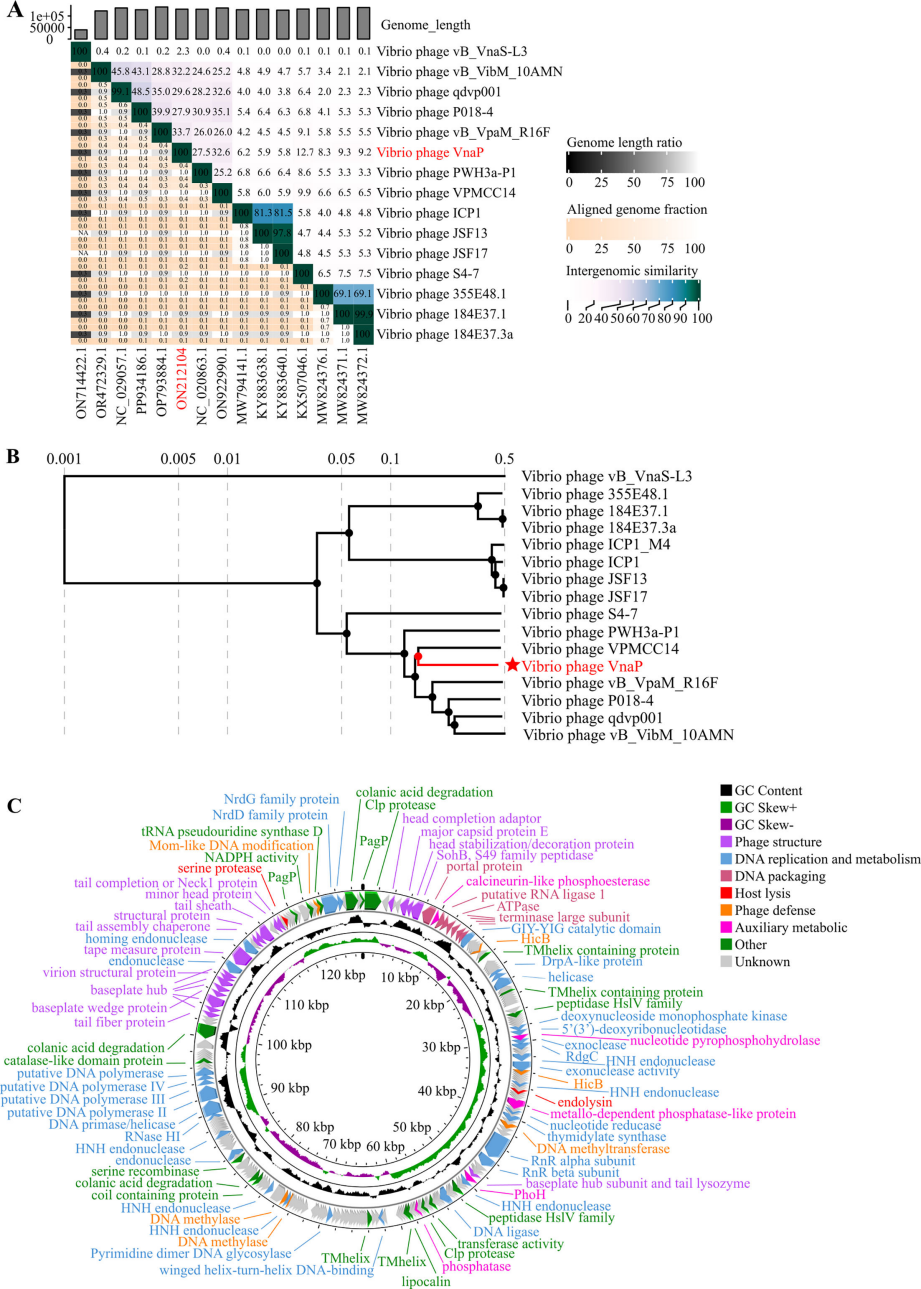
Genomic and phylogenetic characteristics of phage VnaP. (**A**) Phylogenetic tree of VnaP and related phages based on ANI values calculated using VIRDIC. The heatmap displays pairwise ANI percent between genomes. (**B**) Proteomic tree of VnaP generated using ViPTree, illustrating evolutionary distances based on whole proteome comparisons. (**C**) Circular genome map of VnaP depicting predicted ORFs, GC content, and GC skew (from outer to inner). Arrows represent individual ORFs and point the strand direction. Inner rings display GC content and GC skew distribution across the genome.

VnaP possesses a 129,375 bp circular dsDNA genome with 35% GC content. Genome-wide nucleotide comparison using BLASTn identified the closest related genome with ~90% sequence similarity across only 25%–28% of the genome (Table S1). Whole-genome comparative using VIRDIC revealed average nucleotide identity (ANI) values of 33.2%–33.7% between VnaP with its closest related phages (*Vibrio* phage VPMCC14, vB_VpaM_R16F, VibM_10AMN), substantially below the 70% genus-level thresholds (Fig. 2A) (33). Genome synteny analysis further showed limited collinearity with numerous rearrangements relative to these phages (Fig. S2). Proteome-based phylogenetic analysis confirmed its distinct evolutionary position, placing VnaP on a separate branch (Fig. 2B). Collectively, these findings support the classification of VnaP as a representative of a putative novel genus within the *Caudoviricetes*.

The VnaP genome encodes 231 open reading frames (ORFs), of which 92 are assigned putative functions (Table S2). In addition to core proteins essential for the viral life cycle (e.g., structural proteins, DNA replication machinery) (Fig. 2C), the genome encodes several auxiliary metabolic genes (AMGs), including those encoding phosphatase: a classical phosphatase (ORF106) and a metallo-dependent phosphatase-like protein (ORF72). These phage-encoded phosphatases may inhibit the host cellular immune response or regulate intracellular signaling pathways to promote viral replication (34). Importantly, biosafety assessments revealed no identical genes for integrases, antimicrobial resistance, virulence factors, or toxins in phage VnaP. Although a putative lysogenic-associated RdgC homolog (ORF53) was detected, lysogeny was not observed under our experimental conditions (Fig. S3), further supporting its potential as a safe biocontrol agent against *Vibrio* infections.

### Bioinformatic and biochemical characteristics of VnaP-derived depolymerase

#### Bioinformatical analysis predicts that ORF193 encodes a highly divergent depolymerase

Putative depolymerase-encoding ORFs in phage VnaP were identified using the DePolymerase Predictor (DePP) tool, which selects candidates based on amino acid composition and physicochemical properties of characterized phage depolymerases (35). This analysis identified ORF193 (DePP score = 0.92) and ORF230 (DePP score = 0.89) as putative depolymerase genes, though the latter was excluded from subsequent experiments due to unsuccessful heterologous expression. Genomic synteny analysis revealed that the genes flanking ORF193 exhibited limited conservation with related phages, underscoring its unique genomic context. Furthermore, BLASTp analysis of the ORF193 amino acid sequence against NCBI non-redundant (nr) database revealed limited sequence homology, with only three protein hits identified (Table S3), none of which possess experimentally verified enzymatic activity. Additionally, similarity searches against experimentally validated entries in the PDB and UniProt databases returned no significant matches (e-value < 0.001). Consistent with the lack of sequence-level homology, conserved domain analysis using InterProScan failed to detect any known functional domains within the ORF193 sequence, suggesting that it may present a novel depolymerase architecture with previously uncharacterized functional motifs.

**Fig 3:**
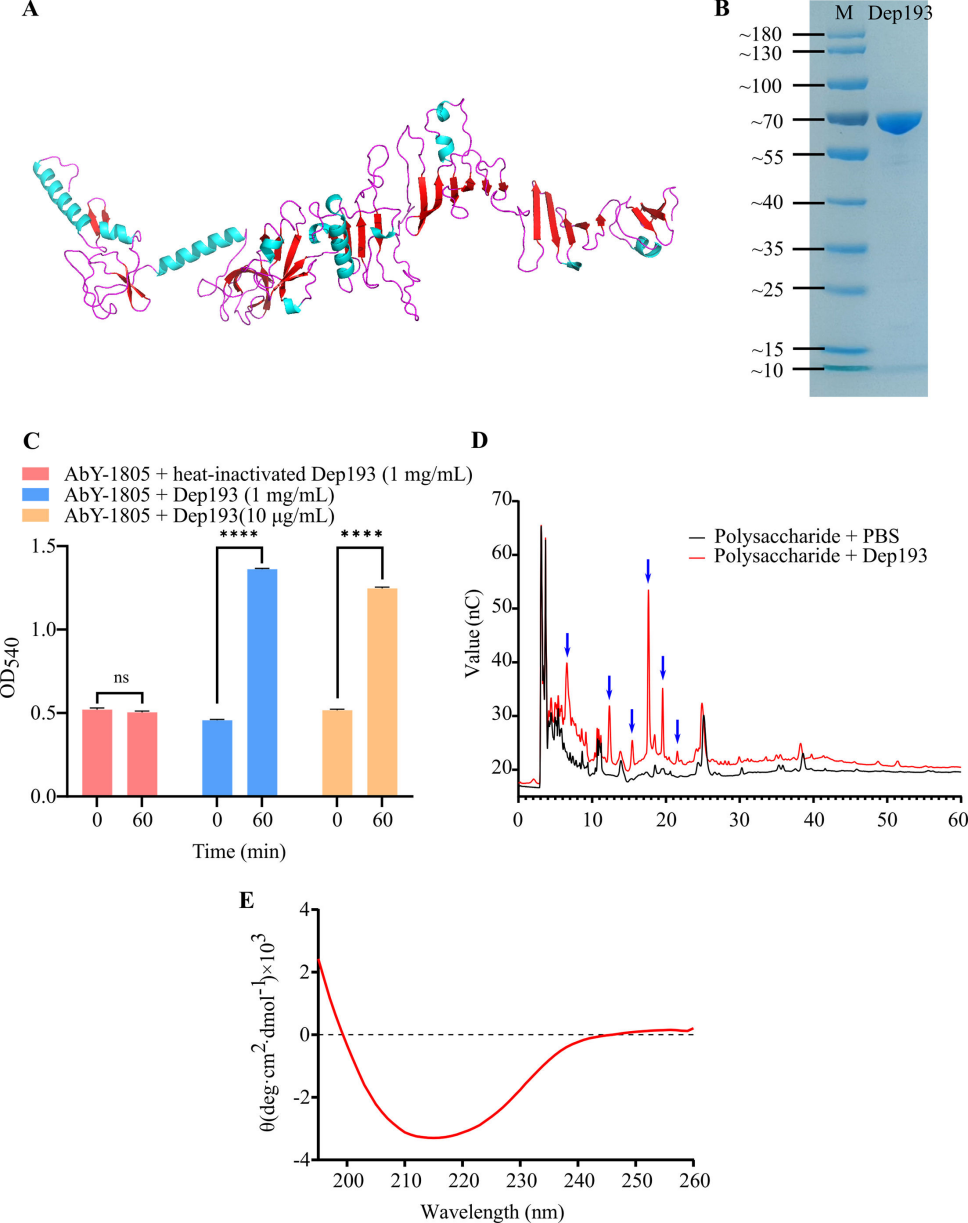
Structural and functional characterization of Dep193. (**A**) Predicted three-dimensional structure of Dep193 generated using AlphaFold3. (**B**) SDS-PAGE analysis of Purified recombinant Dep193. (**C**) Reducing sugars produced from polysaccharide degradation were quantified using the dinitrosalicylic acid (DNS) method following incubation with Dep193 or heat-inactivated enzyme (control). Data represent mean ± SD from three independent experiments (ns, not significant, **** *P* < 0.0001). (**D**) Ion chromatography profiles of host polysaccharides before and after Dep193 treatment. Blue arrows indicate oligosaccharide peaks with notable significant differences between treated and untreated samples. (**E**) Circular dichroism spectrum of Dep193 showing characteristic β-sheet-rich secondary structure.

To further investigate its structural features, a structural model of the ORF193-encoded protein was generated using AlphaFold3 (Fig. 3A, ipTM = 0.61). Subsequent structural homology searches using FoldSeek against the PDB and AlphaFold databases revealed no close structural homologs, with all matches falling below established threshold for structural similarity (TM-score < 0.5, RMSD > 2 Å, Table S4) (36), with detailed structural alignments presented in Fig. S4.

### ORF193 encodes a protein (Dep193) with potent polysaccharide degrading activity

Protein encoded by ORF193 designated Dep193 was predicted to be a 603-amino-acid protein with a molecular weight of 65.2 kDa. Following heterologous expression and purification (Fig. 3B), its enzymatic activity was assessed using polysaccharide extracted from *V. natriegens* AbY-1805 as substrates. DNS assay demonstrated significant polysaccharide degradation, with OD_540_ values increased significantly from 0.5 to 1.3 (ΔA_540_ = 0.8, *P* < 0.001) in Dep193-treated samples compared with negligible activity in heat-inactivated controls (Fig. 3C). Further analysis using ion chromatography revealed a >5-fold increase in peak intensities at several retention times of 6.54, 12.32, 15.43, 17.64, 19.51, and 21.56 min in Dep193-treated samples (Fig. 3D). These peaks likely correspond to lower-molecular-weight products derived from high-molecular-weight polysaccharides, providing additional evidence for Dep193’s depolymerase activity (37). Moreover, UV detection showed no significant increase in the characteristic absorbance at 235 nm in Dep193-treated samples (*P* > 0.05, Fig. S5), indicating an absence of lyase activity and suggesting that Dep193 functions primarily as a hydrolase.

### Secondary structure characterization of Dep193

Secondary structure predictions using PSIPRED indicated that Dep193 is a β-sheet-rich protein (Fig. S6), a structural feature commonly observed in polysaccharide-degrading enzymes of viral and microbial origin (38–40). This prediction was supported by circular dichroism (CD) spectroscopic analysis, which showed that Dep193 adopts a well-folded conformation, characterized by a positive peak near 195 nm and a negative minimum around 215 nm (Fig. 3E), spectral features indicative of β-sheet predominance. Further deconvolution of the CD spectrum using the CONTINLL algorithm estimated the secondary structure composition of 12.2% α-helix, 41.7% β-sheet (comprising 5.4% parallel and 36.3% antiparallel), 19% β-turn, and 22% random coil.

### Synergistic antibacterial activity of phage VnaP and Dep193 against *Vibrio* species

**Fig 4:**
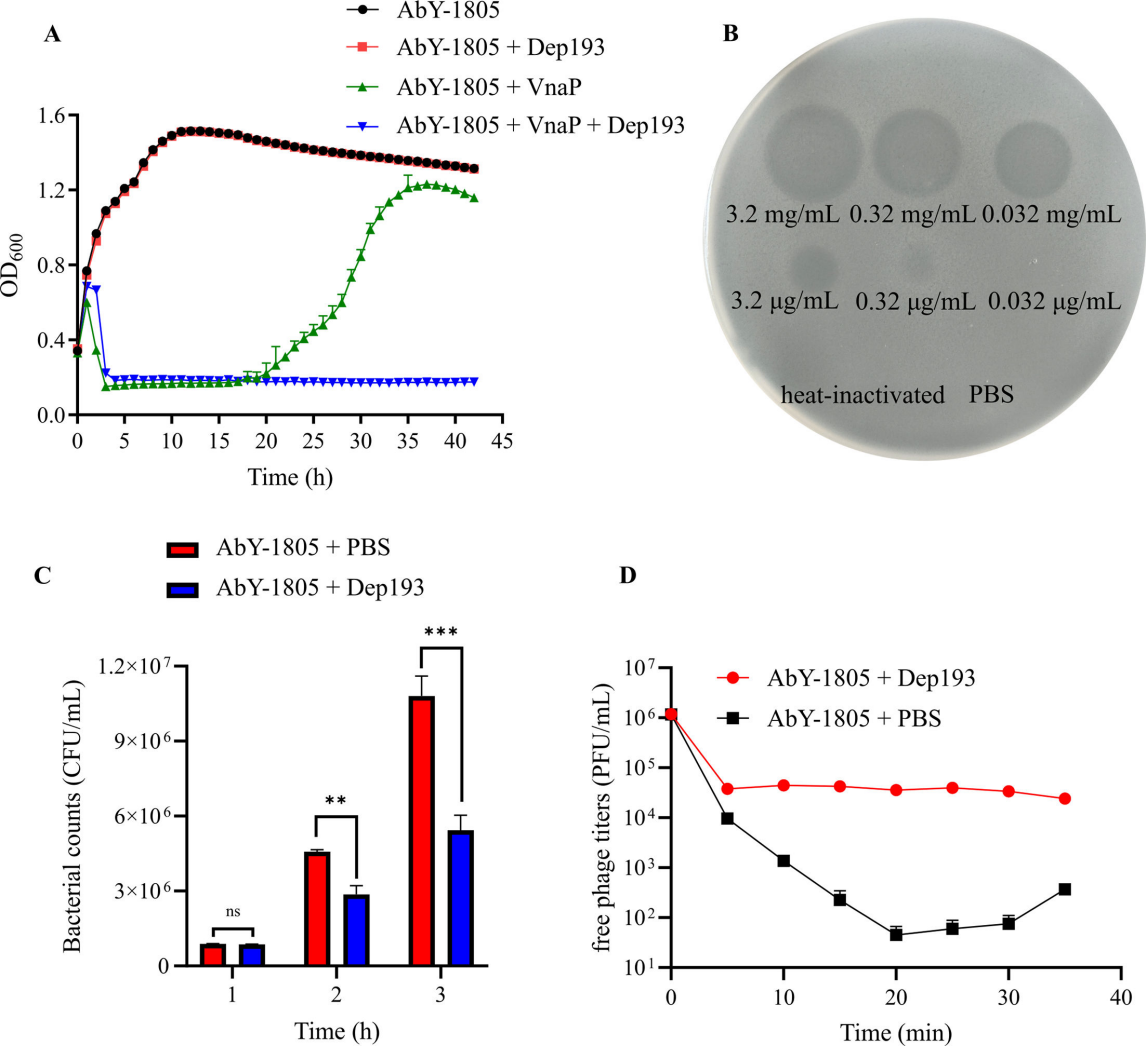
Antibacterial characterization of Dep193. (**A**) Growth curve of *V. natriegens* AbY-1805 under treatment with phage VnaP, Dep193, and their combination. (**B**) Spot assay demonstrating antibacterial activity of Dep193 on *V. natriegens* AbY-1805 lawns. (**C**) Time-course analysis of bacterial viability in *V. natriegens* cultures (~10^5^ CFU/mL initial inoculum). Data represent mean ± SD from three independent experiments (ns, not significant, ** *P* < 0.01, *** *P* < 0.001). (**D**) Phage adsorption kinetics of VnaP on *V. natriegens* AbY-1805 in the presence or absence of Dep193 (1 mg/mL). Data are mean ± SD of three independent experiments.

Phage VnaP treatment robustly suppressed bacterial growth, reducing the OD_600_ from 0.60 to 0.15 within the first 3 h (Fig. 4A). However, its efficacy declined after 20 h, with bacterial regrowth observed thereafter. In parallel, spot assays revealed that Dep193 exhibits antibacterial activity, forming visible clearing zones on bacterial lawns at concentrations as low as 0.32 μg/mL (Fig. 4B). Despite this, Dep193 alone did not significantly reduce turbidity (OD_600_) when applied to high-density, log-phase *V. natriegens* AbY-1805 cultures (~10^8^ CFU/mL). In contrast, when applied to lower-density cultures (~10^5^ CFU/mL), Dep193 (1 mg/mL) significantly reduced viable cell counts, yielding 5.4 × 10^6^ CFU/mL compared with 1.1 × 10^7^ CFU/mL in untreated controls (Fig. 4C). Notably, the combination of Dep193 and VnaP completely inhibited bacterial growth (OD_600_ < 0.2) throughout the entire 40-h incubation period (Fig. 4A). Furthermore, Dep193 exhibited antibacterial activity against *V. diabolicus* YN5, *V. parahaemolyticus* WY7, *V. alginolyticus* SQ20, and *V. atypicus* BR18, consistent with the host range of phage VnaP (Table 2). Similarly, the combination of Dep193 (1 mg/mL) and VnaP completely inhibited growth of *V. atypicus* BR18 and *V. diabolicus* YN5 over 40 h (Fig. S7), underscoring the synergistic potential of this combination for controlling multiple *Vibrio* pathogens.

To further elucidate the mechanism underlying this synergy, we investigated the impact of Dep193 on the phage adsorption. The results revealed that in the absence of Dep193, VnaP rapidly adsorbed to host cells, with free phage titers in the supernatant decreasing from 1.1 ×10^6^ to 45 PFU/mL within 20 min (Fig. 4D). In contrast, the exogenous addition of Dep193 markedly impaired adsorption, with free phage titers nearly 1,000-fold higher than those in untreated controls at the 20-min time point and remaining stable through the 35-min experiment. These results indicate that Dep193 effectively inhibits the initial attachment of Vnap to bacterial cells.

### Biofilm inhibition and degradation capabilities of VnaP, Dep193, and their combination

**Fig 5:**
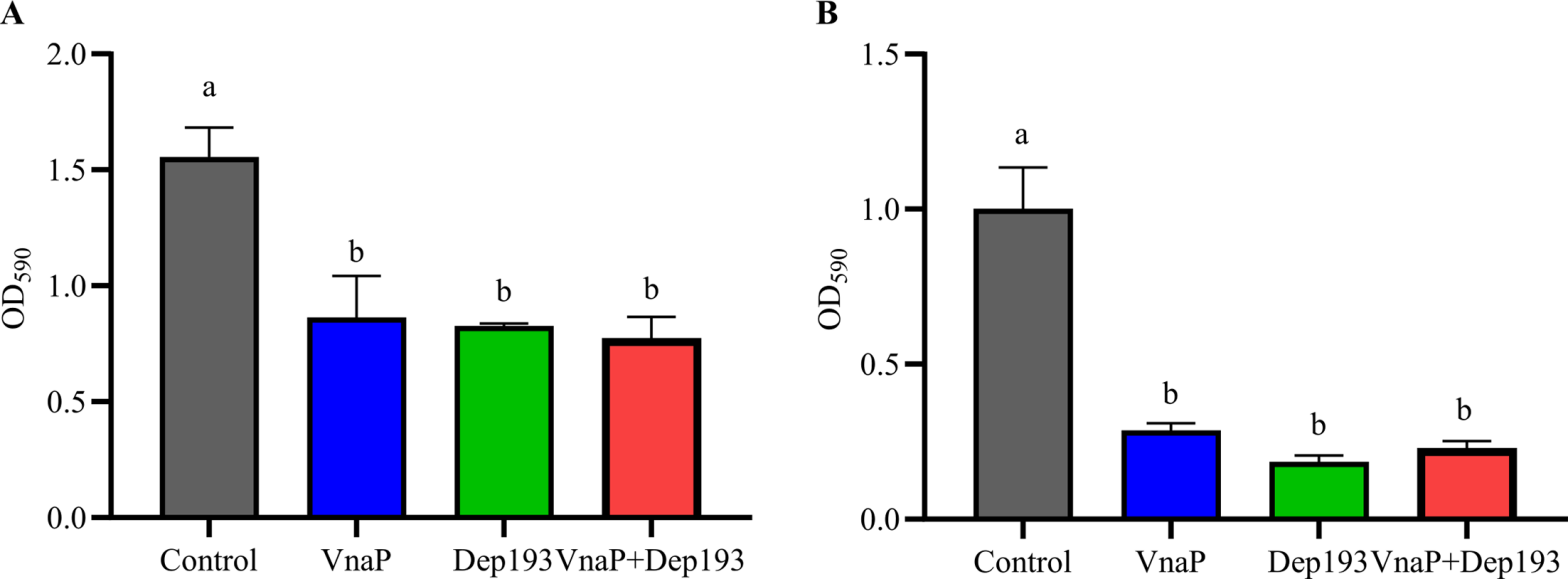
Antibiofilm activity of phage VnaP, Dep193, and their combination against *V. natriegens* AbY-1805. (**A**) Inhibition of biofilm formation. (**B**) Disruption of pre-formed mature biofilms. Data represent the mean ± SD (*n* = 3). Different letters represent significant statistical differences (*P* < 0.05) between groups.

Crystal violet (CV) assays were used to evaluate the antibiofilm activities of VnaP, Dep193, and their combination against *V. natriegens* AbY-1805. All treatments significantly inhibited biofilm formation, reducing OD_590_ values from 1.55 in the untreated control to 0.42–0.50, corresponding to a 67%–72% reduction (*P* < 0.05) (Fig. 5A). Similarly, they markedly disrupted mature biofilms, decreasing OD_590_ values from 1.00 to 0.18–0.29, representing a 71%–82% reduction (*P* < 0.05) (Fig. 5B). These results demonstrate that both phage VnaP and depolymerase Dep193 exhibit strong biofilm-inhibitory and -degradative activities on *V. natriegens* AbY-1805, either alone or in combination, underscoring their potential for controlling *Vibrio* biofilms.

## DISCUSSION

*Vibrio* species are major aquaculture pathogens, leading to substantial economic losses worldwide. Phage-encoded depolymerases, which degrade bacterial polysaccharides, can reduce virulence and disrupt biofilms, making them promising candidates for antibacterial therapy. However, despite their apparent abundance in *Vibrio* phages, depolymerases targeting *Vibrio* spp. remain largely uncharacterized.

In this study, we isolated VnaP, a novel lytic phage infecting *V. natriegens* AbY-1805 and four other *Vibrio* strains. The formation of halo zones in phage plaques on *V. natriegens* AbY-1805 lawns suggested that VnaP encodes a polysaccharide depolymerase (31). Genomic analysis identified ORF193 as the candidate depolymerase gene. However, unlike the majority of experimentally validated phage depolymerases targeting *Klebsiella* and *Acinetobacter* species, which possess recognizable catalytic or structural domains (34 of 40 surveyed in UniProt and PDB), Dep193 shows no significant sequence or structural homology to known depolymerases and lacks any identifiable functional motifs, highlighting its novelty. A broader bioinformatic analysis of cultured *Vibrio* phage-encoded depolymerases revealed that while putative depolymerase genes are prevalent in cultured *Vibrio* phage genomes, only a small fraction (30.7%, 66/215) possesses identifiable functional domains, such as carbohydrate esterases or pectate lyase superfamily motifs. This observation highlights a critical limitation in current domain-based annotation methods and underscores the necessity for biochemical validation to characterize novel depolymerases. Phylogenetic analysis showed that depolymerases from *Vibrio* phages form a distinct evolutionary lineage, within which Dep193 represents the first experimentally confirmed member (Fig. S8). This finding broadens the current understanding of *Vibrio*-associated depolymerase diversity and offers a valuable reference for identifying previously uncharacterized depolymerases (11, 41).

Consistent with previous reports that phage-derived depolymerases generally reduce bacterial growth rather than causing outright cell death (42–44), LIVE/DEAD staining demonstrated that *V. natriegens* AbY-1805 cells treated with Dep193 exhibited decreased viability compared with untreated controls while maintaining membrane integrity (Fig. S9). Accordingly, the lytic zones observed on agar plates likely reflect capsule-dependent growth inhibition rather than cell lysis (45). The minimum concentration at which depolymerases exhibit inhibitory effects on agar plates varies among enzymes. For example, depolymerases targeting bacteria, such as *Klebsiella pneumoniae* and *Escherichia coli*, have been reported to act effectively at 0.1 to 0.5 μg/mL (42, 46–48). In this study, Dep193 produced clear zones at 0.32 μg/mL, within the range reported for other phage-derived depolymerases, indicating comparable enzymatic potency. By contrast, this inhibitory effect was less pronounced in liquid culture, as measured by bacterial enumeration and optical density (Fig. 4A and C). At lower concentrations (e.g., 10 μg/mL), Dep193 exhibited limited inhibitory effects, likely due to rapid bacterial proliferation under favorable conditions (49) and differences in capsular polysaccharide expression between planktonic (liquid) and surface-associated (agar) growth states (50–52). Based on these observations, a concentration of 1 mg/mL was selected for subsequent growth curve and antibiofilm assays, where Dep193 demonstrated significant antibacterial activity and biofilm-modulating properties, both preventing biofilm formation and degrading mature biofilm structures. Importantly, Dep193 exhibited synergistic effects when combined with phage VnaP, with the combination completely inhibiting the growth of two additional *Vibrio* species (*V. atypicus* BR18 and *V. diabolicus* YN5). This broad-spectrum efficacy against multiple *Vibrio* species highlights the therapeutic potential of the Dep193-VnaP combination as a strategy for controlling polymicrobial *Vibrio* infections in aquaculture environments.

A major challenge in phage therapy is the emergence of phage-resistant bacteria, which has been addressed through approaches, such as phage cocktails or phage-antibiotic combinations (53–55). While these strategies can extend host range and delay resistance development, complementary approaches are required to optimize therapeutic outcomes, particularly against biofilm-embedded or polysaccharide-shielded pathogens (20, 21). Phage-encoded depolymerases have emerged as promising complementary agents, capable of degrading biofilm matrices that impede phage access (20, 21) or removing surface polysaccharides to expose secondary receptors and facilitate infection (14, 22). In this study, Dep193 exhibited modest antibacterial activity on its own but substantially enhanced the efficacy of phage VnaP by preventing bacterial regrowth. Interestingly, adsorption assays revealed that Dep193 treatment reduced phage adsorption efficiency relative to untreated controls (Fig. 4D), implying that Dep193 may diminish available receptor-binding sites rather than promoting infection through secondary receptor exposure. This unexpected behavior suggests that the synergy between Dep193 and VnaP operates through a mechanism different from previously reported depolymerase-phage interactions (45). Future investigations should focus on molecular domain analysis of Dep193, identification of its host receptor, and receptor complementation experiments to comprehensively characterize this unique enzyme and to experimentally validate the competitive inhibition hypothesis of Dep193 in phage adsorption. Notably, Dep193 maintained antibacterial activity against seven of ten selected phage-resistant strains (Fig. S10), indicating that its synergistic effect may stem from either direct suppression of the bacterial population, thereby limiting resistance emergence, or from metabolic stress induced by Dep 193 that weakens bacterial physiology and enhances phage susceptibility (49). These results support the utility of depolymerases as effective adjuncts to phage therapy and highlight their potential in developing more robust antimicrobial strategies for combating *Vibrio* infections.

## MATERIALS AND METHODS

### Genomic and phylogenetic characterization of *Vibrio* phage depolymerases

Cultured and uncultured *Vibrio* phage genomes were retrieved from the RefSeq v210 and IMG/VR v4 database (56), respectively. Genome completeness and contamination were assessed using CheckV v1.0.1, and only high-confidence *Caudoviricetes* phage genomes (completeness ≥ 90%, and contamination < 5%) were retained for further analysis. ORFs were predicted using Prodigal v2.6.3 (57) in metagenomic mode, and the resulting protein sequences were screened for putative depolymerases using the DePolymerase Predictor (DePP) v1.0.0. Candidate genes were selected using an empirical DePP score threshold of 0.90, established by benchmarking the tool against experimentally validated phage depolymerases from the public database (UniProt and PDB) (Table S5).

### Phage isolation and purification

The host strain *V. natriegens* AbY-1805 was isolated from diseased abalone (*Haliotis discus hannai Ino*) tissue (58). The bacteria were cultured in RO medium (1% tryptone, 1% yeast extract, 1% sodium acetate in artificial seawater, pH 7.8-8.0) at 28°C with shaking. Upon reaching log-phase growth (OD_600_ ≈ 0.60), coastal seawater samples from Qingdao, China, were filtered through 0.22-μm sterile membranes and added to the bacterial culture at a 10% (v/v) ratio. The mixed culture was incubated at 28°C with shaking, and samples were collected daily for seven consecutive days and filtered for phage detection. Phage presence was confirmed using double-agar overlay plaque assay (59). Briefly, 1 mL of filtrate was mixed with 1 mL of log-phase bacterial culture, incubated for 30 min at 28°C, then combined with 4 mL molten RO soft agar (0.5%) and overlaid onto solidified RO agar plates (1.5%). Individual plaques were selected and eluted in SM buffer, with this purification procedure repeated five times to obtain pure phage isolates.

### TEM

Phage morphology was examined by TEM (54). Briefly, phage suspension (1 mL) was concentrated using a 30-kDa ultrafiltration membrane and washed twice with SM buffer. For visualization, an aliquot (10 μL) of the concentrated suspension was deposited onto a carbon-coated 200-mesh copper grid and air-dried at room temperature. The sample was then negatively stained with 2% uranyl acetate for 3 min. Microscopy examination was performed using a Hitachi-7800 TEM operated at an accelerating voltage of 80 kV.

### Host range

A spot assay was performed to determine the host range of phage Vnap using 35 bacterial strains from five genera, all of which were isolated from diseased shrimp and fish in our laboratory (60). Briefly, 1 mL of log-phase tested bacterial culture was mixed with 5 mL of molten RO soft agar and overlaid onto the solidified RO agar plate. Subsequently, 5 μL of serially diluted phage suspensions were spotted onto the bacterial lawn and incubated at 28°C overnight. Bacterial sensitivity to phage infection was assessed based on the formation of clearing zones.

### One-step growth curve

The one-step growth curve for VnaP was determined following established protocols with minor modifications (61–63). Briefly, a 1 mL aliquot of log-phase *V. natriegens* AbY-1805 culture (OD_600_ ≈ 0.60, ~10^8^ CFU/mL) was infected with 100 μL of VnaP phage lysate (10^6^ PFU/mL), achieving a multiplicity of infection (MOI) of approximately 0.001. After 10-min adsorption at 28°C, unadsorbed phages were removed by centrifugation (5,000 × *g*, 3 min). The cell pellet was washed three times with fresh RO medium. The washed pellet was resuspended in 1 mL of RO medium. From this resuspension, 100 µL was immediately serially diluted to determine the number of initial infected cells using the double agar overlay assay. Concurrently, 20 μL of this resuspension was diluted into 20 mL of fresh RO medium. The diluted culture was incubated at 28°C with shaking. Samples were collected at 5-min intervals over a 60-min period. Each sample was immediately filtered through 0.22-μm membranes, and free phage titers were quantified using the double-layer agar method. The burst size was calculated as the ratio of the final phage titers to the initial infected bacterial cells.

### Genomic sequencing and analysis

Phage genomic DNA was extracted and purified as previously described (64). DNA sequencing was performed on the Illumina Hiseq 2500 platform by Oebiotech Co. (Qingdao, China). Sequencing reads were assembled using SPAdes Genome Assembler v.3.14.1 (65). Genome completeness and contamination removal were performed using CheckV v1.0.1 (66). The genome type was verified by PCR amplification using the following primers: VnaP-CF (5′-GCGAAAGATAAATTGCAGTGC-3′) and VnaP-CR (5′-GACAAGAAGTGGATTAGCCTTC-3′) (54). ORFs were predicted using GeneMarks and annotated manually through BLASTP searches (https://www.ncbi.nlm.nih.gov/) against the NCBI non-redundant (nr) protein database with an e-value threshold of 10^−5^. Functional genomic elements, including tRNAs, virulence factors, and antimicrobial resistance genes, were identified using PhageScope (67). The annotated genome was visualized using Proksee server (68). Comparative genomic analysis included whole-genome nucleotide comparisons using BLASTN against the NCBI nucleotide collection (nt) database and proteomic comparisons using VipTree 4.0 (69). Average nucleotide identity was calculated using VIRDIC (33).

### Bioinformatic analysis of Dep193

Putative depolymerase genes were identified using DePolymerase Predictor (35), a machine learning-based tool with 90% classification accuracy by analyzing amino acid composition and key physicochemical properties. Conserved domain of candidates depolymerases was analyzed using InterProScan (https://github.com/ebi-pf-team/interproscan). The three-dimensional structure of Dep193 was predicted using Alphafold3 server (70) and visualized using PyMOL (http://www.pymol.org/pymol). The FoldSeek server was used to search structural homology proteins against PDB and AlphaFold database (71). To strengthen functional assignment, all DePP-predicted candidates from cultured *Vibrio* phages were cross-checked using BLASTp against the NCBI non-redundant database and examined for conserved domains with InterProScan, retaining only sequences with significant homology and identifiable functional domains for further analysis. The identified depolymerase genes, together with experimentally validated phage depolymerase genes retrieved from PDB and UniProt database (72), were subjected to redundancy reduction using CD-HIT v4.8.1 (73) with a sequence identity threshold of 95% (parameters: -c 0.95). The resulting non-redundant representative sequences were subsequently used for phylogenetic tree construction. Multiple sequence alignment was performed using MAFFT v7.471 (74) (--maxiterate 1000—localpair), and poorly aligned regions were removed using trimAl v1.4.rev15 (-gt 0.5) (75). A maximum-likelihood phylogenetic tree was generated using IQ-tree v.2.0.3 (76) under the best-fit model (Blosum62+F+R5) with 1,000 ultrafast bootstrap replicates. The resulting phylogenetic tree was visualized using iToL v6 (77). The trimmed amino acid alignment is available in the supplemental material.

### Heterologous expression and purification of Dep193

The ORF193 sequence encoding the depolymerase Dep193 was amplified by PCR using the following primer pair: 193-HA-F (5′-accatcatcaccacagccagACTTACCCAGCAACTAATAG-3′) and 193-HA-R (5′-gcattatgcggccgcaagctTTAAACTACTACACCAGTTG-3′), where the lowercase sequences representing homology arms for subsequent Gibson assembly. The pRSF-Duet-1 vector was linearized using *BamHI* and *HindIII* restriction enzymes. Following purification, the PCR product and linearized vector were assembled using 2× MultiF Seamless Assembly Mix (ABclonal) to generate the recombined plasmid pRSF-Duet-1-ORF193. After sequence verification, the plasmid was transformed into *E. coli* BL21 (DE3) cells, and the recombinant strain was cultured in LB medium supplemented with kanamycin (50 μg/mL) at 37°C until reaching log phase. Protein expression was induced by the addition of 0.5 mM isopropyl-β-D-1-thiogalactopyranoside, followed by incubation at 16°C for 16 h. After induction, bacterial cells were harvested by centrifugation at 5,000 × *g* for 30 min at 4°C, then resuspended in PBS buffer (137 mM NaCl, 2.7 mM KCl, 10 mM Na_2_HPO_4_, 1.8 mM KH_2_PO_4_, pH 7.0). Cell lysis was performed by sonication (3-s pulses with 4-s intervals) on ice for 1 h, followed by centrifugation at 8,000 × *g* for 30 min at 4°C to remove cell debris. The His-tagged Dep193 protein was purified from the clarified supernatant using an ÄKTA pure chromatography system with a Ni-NTA column, following established protocols for affinity chromatography (78). Elution was performed using a gradient of PBS buffer containing imidazole (pH 7.0), and fractions containing the target protein were pooled. The purity of the recombinant Dep193 protein was assessed by 8%–10% SDS-PAGE, followed by visualization using Coomassie Brilliant Blue staining. The concentration of purified protein was determined spectrophotometrically using a Nanodrop and stored at −80°C for further analysis.

### CD analysis

CD spectroscopy was performed using a Chirascan V100 spectropolarimeter. CD measurements were constructed at 20°C using a 0.05-cm path length cuvette with protein samples at a concentration of 0.5 mg/mL in 10 mM PBS buffer (pH 7.0). Spectra were recorded from 260 to 195 nm with an integration time of 0.5 s per wavelength. All spectra represent the average of three independent scans and were baseline-corrected against a PBS buffer blank (10 mM, pH 7.0). The observed ellipticity (θ_obs) was converted to mean residue ellipticity [θ] using the formula: [θ] = θ_obs × M_w / (10 × c × L × n), where M_w is the molecular weight (Da), c is the protein concentration (mg/mL), l is the path length (cm), and n is the number of amino acid residues. Secondary structure analysis was performed using the CDNN deconvolution algorithm.

### Polysaccharide degradation activity of Dep193

To directly assess Dep193 depolymerase activity on *V. natriegens* AbY-1805 and its relationship to antibacterial effects, capsular polysaccharides extracted from *V. natriegens* AbY-1805 (see the supplemental methods) (79) were used as substrates in two complementary assays.

### 3,5-DNS method

The 3,5-DNS assay, a rapid and sensitive method for detecting reducing sugars released during polysaccharide cleavage, was used to provide preliminary measure of depolymerase activity (80). In brief, purified Dep193 and heat-inactivated Dep193 (100°C for 10 min) were separately incubated with the extracted polysaccharide at a final concentration of 10 μg/mL and 1 mg/mL. Control reactions included PBS mixed with either polysaccharide or Dep193 alone. All reaction mixtures were incubated at 28°C for 1 h. After incubation, DNS reagent was added to each mixture at a 2:1 volume ratio, and the samples were heated at 100°C for 5 min. The formation of reducing sugars was quantified by measuring absorbance at 540 nm.

### Product characterization by ion chromatography

To further confirm depolymerase activity and visualize degradation products, oligosaccharide products from enzyme-substrate reaction mixtures were analyzed using ion chromatography. Samples from reducing sugar quantification experiments were analyzed using a Dionex ICS-5000^+^ Ion Chromatography System (Thermo Fisher Scientific Inc., USA) equipped with a Dionex CarboPac PA200 analytical column (3 × 250 mm). The mobile phase consisted of three components: (A) 250 mM NaOH, (B) H_2_O, and (C) 1 M sodium acetate (NaAc). The gradient elution program was as follows: 0–35 min, 40% A/53% B/7% C; 35–110 min, 40% A/40% B/20%C; 110–115 min, 25% A/40% B/35% C; 115–120 min, 10% A/40% B/50% C; 120–125 min, 40% A/40% B/20% C; 125–130 min, 40% A/53% B/7% C. Samples (5 μL) were injected at a flow rate of 0.3 mL/min. As the detailed composition of polysaccharides extracted from *V. natriegens* AbY-1805 has not been fully characterized, the use of reference standards or known oligosaccharide markers was not feasible. Chromatographic profiles of samples incubated with Dep193 were therefore compared with untreated polysaccharide samples, and the emergence of new peaks corresponding to lower-molecular-weight products served as evidence of polysaccharide degradation (37).

### Antibacterial activity assessment

The antibacterial activity of Dep193 was evaluated using both spot assays and liquid culture assays. For the spot assay, purified Dep193 (3.2 mg/mL) was serially diluted 10-fold in PBS (pH 7.0), and 5 μL of each dilution was spotted onto bacterial lawn plates of *V. natriegens* AbY-1805, prepared as previously described. Controls included PBS (pH 7.0) and heat-inactivated Dep193 (100°C for 10 min). Plates were incubated overnight at 28°C, and antibacterial activity was assessed by measuring the diameter of halo zones, indicative of bacterial lysis.

For the liquid assay, 10 μL of log-phase *V. natriegens* AbY-1805 culture (OD_600_ ≈ 0.60) was inoculated into 1 mL of RO liquid medium supplemented with either Dep193 (final concentration of 1 mg/mL) or PBS (control). One hundred microliter samples were collected at 0, 1, and 2 h post-treatment for bacterial enumeration. In parallel, bacterial samples collected at 2 h post-treatment were stained using LIVE/DEAD BacLight Bacterial Viability Kits (Thermo Fisher Scientific) following the manufacturer’s protocol. The stained samples were then visualized using a confocal laser scanning microscopy (LSM 900, 63× oil objective) at excitation/emission (Ex/Em) wavelengths of 480/500 nm for live cells (SYTO 9) and 490/635 nm for dead cells (propidium iodide).

Additionally, the combined antibacterial activity of Dep193 and phage VnaP was assessed. Log-phase bacterial cultures were incubated with the following treatments: (i) phage VnaP (MOI = 0.1), (ii) phage VnaP + Dep193 (MOI = 0.1 and final concentration of 1 mg/mL), (iii) Dep193 (final concentration of 1 mg/mL), and (iv) PBS (control). Bacterial growth was monitored by measuring optical density at 600 nm at 1-h interval.

### Antibiofilm activity assessment

The antibiofilm activities of VnaP, Dep193, and their combination were evaluated using the CV quantification assays with minor modifications to established protocols (81, 82). For the biofilm inhibition assay, 80 μL of RO medium and 10 μL log-phase bacterial suspension (OD_600_ ≈ 0.6) were added to 96-well plates. Then 10 μL of PBS (control), phage lysate in PBS (1 × 10^8^ PFU/mL), Dep193 (final concentration of 1 mg/mL), or a combination of phage lysate and Dep193 was added separately to each well. Plates were incubated at 28°C for 24 h, after which planktonic cells were removed, and the wells were washed twice with PBS. The remaining biofilms were fixed with 100 μL of methanol for 15 min, stained with 100 μL of 0.1% (w/v) CV for 10 min, and rinsed three times with distilled water. The stain was solubilized with 100 μL of 33% (v/v) glacial acetic acid, and biofilm biomass was quantified by measuring absorbance at 590 nm.

For the biofilm degradation assay, mature biofilms were established by incubating 10 μL of log-phase suspension in 90 μL RO medium at 28°C for 24 h. After removing planktonic cells and washing with PBS, biofilms were separately incubated with 100 μL of PBS (control), phage lysate in PBS (1×10^8^ PFU/mL), Dep193 (final concentration of 1 mg/mL), or a combination of phage lysate and Dep193 at the same concentration at 28°C for 6 h. Wells were then washed, stained, and quantified using the crystal violet staining protocol described above.

The biofilm inhibition or degradation percentage (%) was calculated as follows:


$$Percent\;reduction=[(OD_{590\_control}-OD_{590\_treatment})/OD_{590\_control}]\times 100\%$$

### Statistical analysis

All experiments were performed in biological triplicates. Statistical analysis was conducted using GraphPad Prism nine with one-way ANOVA or student’s t test. A *P*-value < 0.05 was considered statistically significant.

## Data Availability

The complete genome sequence of phage VnaP and the protein sequence of Dep193 were deposited at NCBI GenBank under accession numbers ON212104 and UYE94572, respectively.

## SUPPLEMENTAL MATERIAL

File S1 aem.01824-25-s0001.FASTA Amino acid sequences of depolymerases after alignment trimming, which was used for phylogenetic tree construction (see Fig. S8).

File S2 aem.01824-25-s0002.eps Multiple sequence alignment of depolymerases after trimming (conserved amino acid residues are highlighted in color).

Supplemental material aem.01824-25-s0003.docx Supplemental methods, Fig. S1 to S10, and Tables S1 to S5.

ASM does not own the copyrights to Supplemental Material that may be linked to, or accessed through, an article. The authors have granted ASM a non-exclusive, world-wide license to publish the Supplemental Material files. Please contact the corresponding author directly for reuse.
