# Supplementary material for: Mining a vibriophage depolymerase for enhanced pathogen control in aquaculture
Source: Appl Environ Microbiol. 2026 Jan 12;92(2):e01824-25. doi: 10.1128/aem.01824-25 (PMC12915355; doi:10.1128/aem.01824-25)
Supplement: Supplemental material — Supplemental methods, Fig. S1 to S10, and Tables S1 to S5. [file aem.01824-25-s0003.docx]

**Supporting Information for**

**Mining a vibriophage depolymerase for enhanced pathogen control in aquaculture**

Yufei Yue^1, 2^, Jiulong Zhao^1, 2, 3, 4, 5^, Zengmeng Wang^1, 2, 4^, Rui Yin^1, 2, 6^, Yang He^6^, Chengcheng Li^1, 2, 4*^, Yongyu Zhang^1, 2, 3, 4, 5*^

^1^ Qingdao New Energy Shandong Laboratory, Key Laboratory of Biofuels, Shandong Provincial Key Laboratory of Energy Genetics, Qingdao Institute of Bioenergy and Bioprocess Technology, Chinese Academy of Sciences, Qingdao, China

^2^ University of Chinese Academy of Sciences, Beijing, China

^3^ Southern Marine Science and Engineering Guangdong Laboratory (Zhuhai), Zhuhai, China

^4^ Shandong Energy Institute, Qingdao, China

^5^ Laboratory for Marine Biology and Biotechnology, Qingdao Marine Science and Technology Center, Qingdao, China

^6^ Ocean University of China, Qingdao, China

***Correspondence**:

Yongyu Zhang, zhangyy@qibebt.ac.cn; Chengcheng Li, licc@qibebt.ac.cn

**This file includes:**

Materials and Methods

Figures S1–S10

Tables S1–S5

References

Additional supplemental files:

**File S1. Alignment-trimmed FASTA file used for phylogenetic analysis.**

This file contains the amino acid sequences of depolymerases after alignment trimming, which was used for phylogenetic tree construction (Figure S8 in the Supplementary Materials).

**File S2.** **Visualization of trimmed sequence alignment (EPS format).**

This figure shows the multiple sequence alignment of depolymerases after trimming. Conserved amino acid residues are highlighted in color. The visualization was generated using Jalview.

**Materials and Methods**

***Chloroform sensitivity***

Chloroform sensitivity of phage was evaluated by incubating phage lysate with chloroform at final concentrations of 0% (control), 2% and 20% (v/v). The mixtures were vortexed vigorously for 30 s and incubated at room temperature for 30 min. After centrifugation at 60,000 × g for 5 min to remove chloroform, the aqueous phase was spotted onto bacterial lawns of log-phase *Vibrio* *natriegens* AbY-1805.

***Isolation of phage-resistant strains***

Phage-resistant mutants were selected as previously described (1). Briefly, 100 µL of log-phase (OD_600_ ≈ 0.60) *Vibrio natriegens* AbY-1805 culture was spread evenly on RO agar plates to form a uniform bacterial lawn. Subsequently, 10 µL of phage VnaP suspension (~10^8^ PFU/mL) was spotted onto the lawn. Following incubation at 28 °C for 12 h, clear plaques appeared on the plates, within which a few bacterial colonies were observed. These colonies were selected and purified through streak-plating to obtain individual isolates. Each isolate was then tested for phage susceptibility using spot assays, and those that remained insensitive to VnaP were classified as phage-resistant mutants. These strains were verified by 16S rRNA gene sequencing and stored in 0.85% NaCl solution containing 25% glycerol at -80 °C for further analysis.

***Lysogeny test***

Lysogeny of phage VnaP was investigated as previously described (2). Briefly, genomic DNA from phage-resistant mutant strains was analyzed by PCR to detect integrated phage sequences. The wild-type strain served as negative control and phage VnaP genomic DNA served as positive control. PCR amplification was performed using the following primers: Lyso-F (5’- TACCATCGGATAATTTTCGTCC-3’) and Lyso-R (5’- CCAATTCACCTACACCACG-3’).

***Extraction and quantification of bacterial capsular polysaccharide***

Bacterial capsular polysaccharide was extracted from *Vibrio natriegens* AbY-1805 using established protocols with modification (3). Briefly, log-phase bacterial culture (1 L) was centrifuged at 10,000 *×* g for 5 min, and the pellet was washed three times with sterile PBS. Washed cells were resuspended in 10 mL PBS and subjected to hot phenol-water extraction (1:1 v/v water-saturated phenol, 65°C, 20 min). Following three chloroform extractions to remove protein contaminants, the aqueous phase was collected, lyophilized, and stored in PBS at -20°C.

Polysaccharide concentration was determined by the phenol-sulfuric acid colorimetric method. Samples (50 μL) were mixed with 5% (w/v) aqueous phenol (50 μL) and concentrated sulfuric acid (250 μL), incubated at room temperature for 20 min, and absorbance measured at 490 nm. A standard curve was generated using glucose solutions (0.1–1 mg/mL) processed in parallel.

***C4­–C5 unsaturated bonds detection***

To further elucidate the catalytic mechanism of Dep193, host-extracted polysaccharides (1 mg/mL) were treated with either active Dep193 (1 mg/mL) or heat-inactivated Dep193 (1 mg/mL, control) at 30°C for 60 min to enable depolymerase-mediated cleavage. The absorbance of the reaction mixture at 235 nm was measured to monitor the formation of C4–C5 unsaturated bonds, indicative of potential lyase activity (4).

***Phage adsorption assays***

The effect of Dep193 on phage adsorption was evaluated using a quantitative adsorption kinetics assay (5). Briefly, log-phase *V. natriegens* cultures (OD_600_ ≈ 0.6, ~1 × 10^8^ CFU/mL) were co-incubated with phage VnaP (MOI = 0.01) in the presence or absence of Dep193 (1 mg/mL). At 5-min intervals post-infection, samples were collected and immediately filtered through 0.22-μm membranes to remove bacteria and adsorbed phages. The titer of free (unadsorbed) phages in the filtrate was then determined by plaque assay as described above.

**Figures**


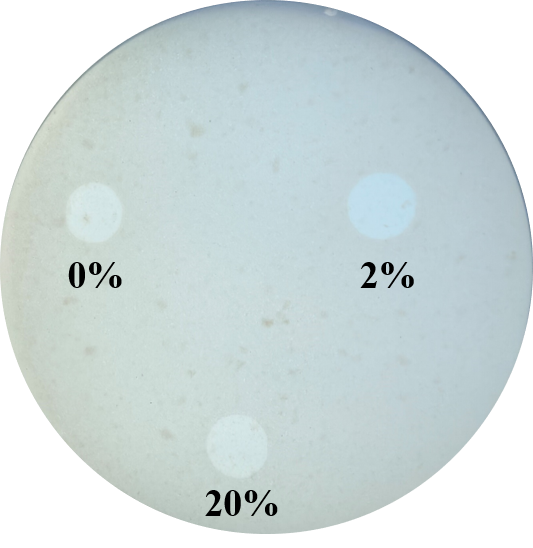


**Fig. S1** **Spot assay demonstrating the chloroform resistance of phage VnaP.**


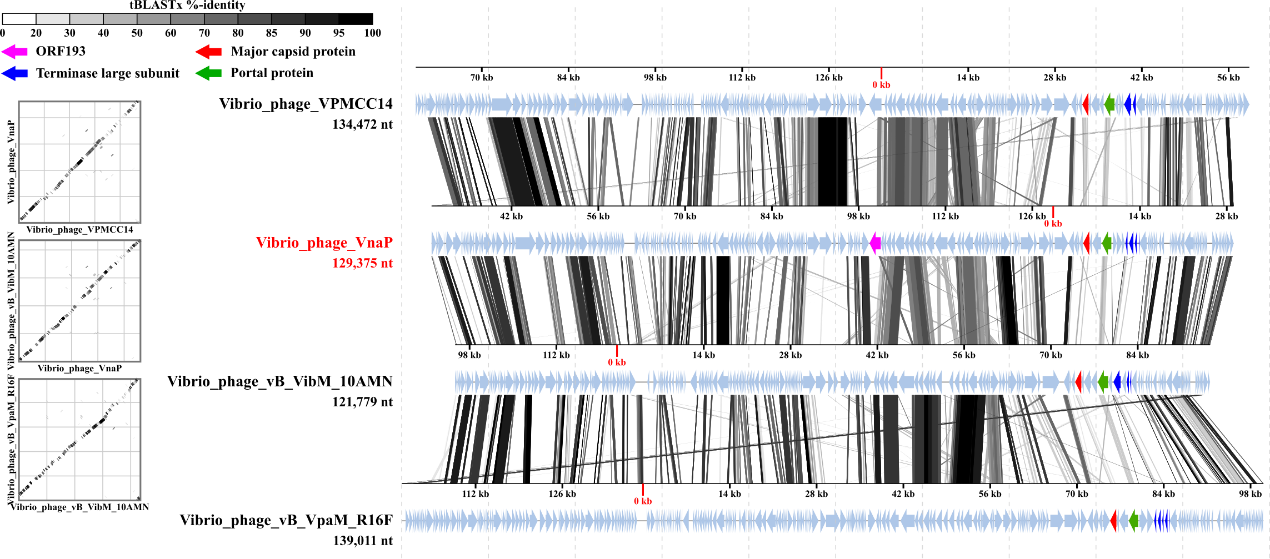


**Fig. S2 Genomic synteny analysis of phage VnaP and its closest relatives.** Colored blocks indicate homologous regions, with sequence identity represented by a 0–100% color scale. ORF193 is highlighted in red.


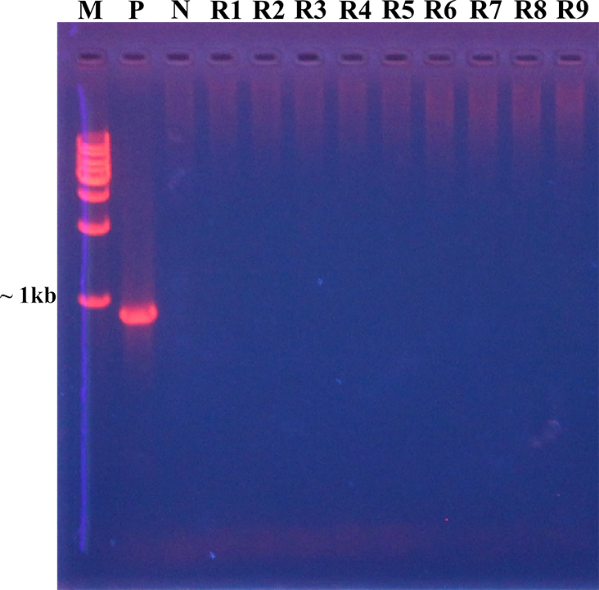


**Fig. S3** **Lysogeny analysis of VnaP in phage-resistant *V. natriegens* AbY-1805 isolates.** Lane M: Molecular weight maker; Lane P: Positive control; Lane N: Negative control; Lane R1–R9: Individual VnaP-resistant isolates. The lack of bands in lanes R1–R9 suggests the absence of lysogenic integration by VnaP.


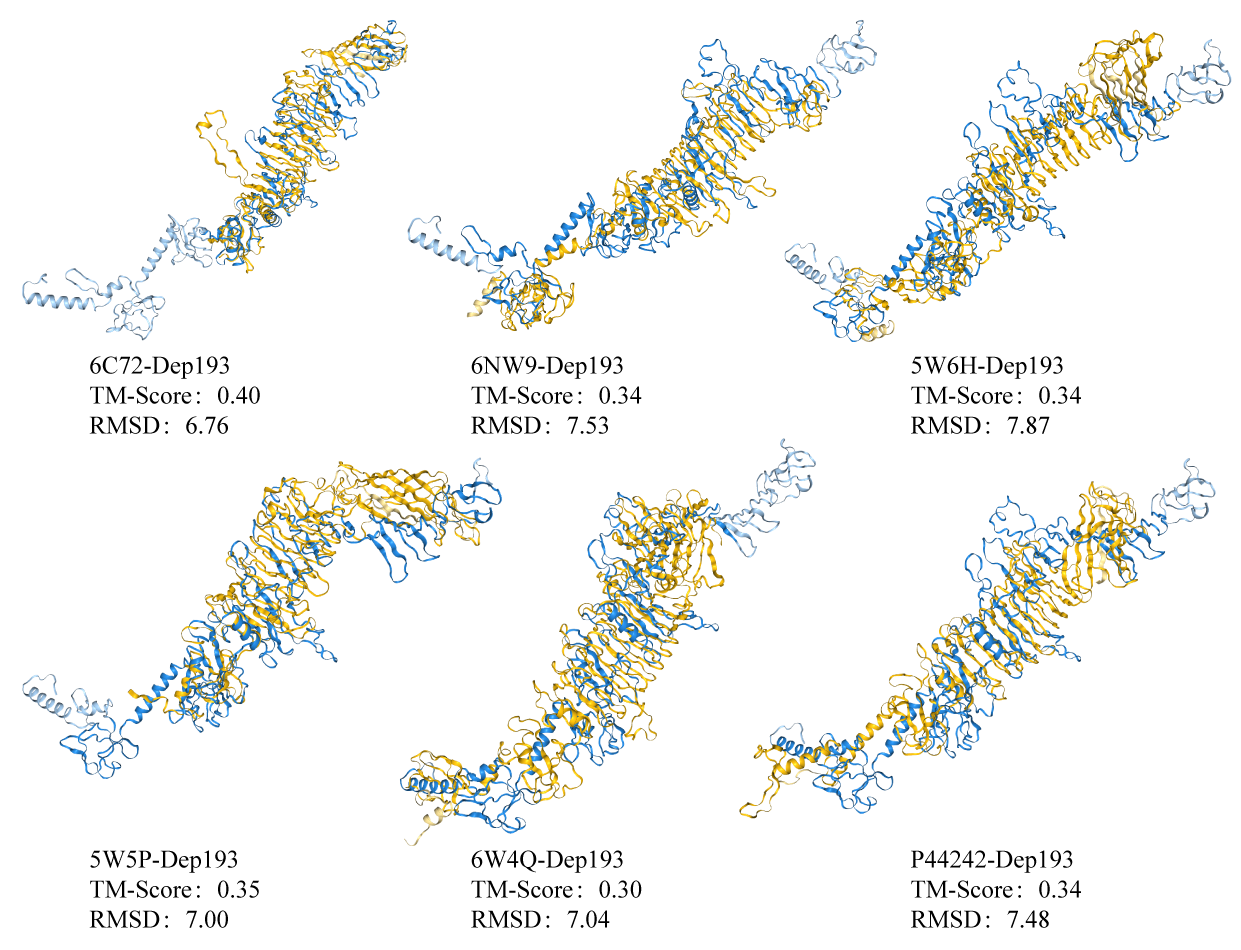


**Fig. S4** **Structural alignment of Dep193 (blue) with its closest PDB homologs (yellow) based on top FoldSeek matches.**


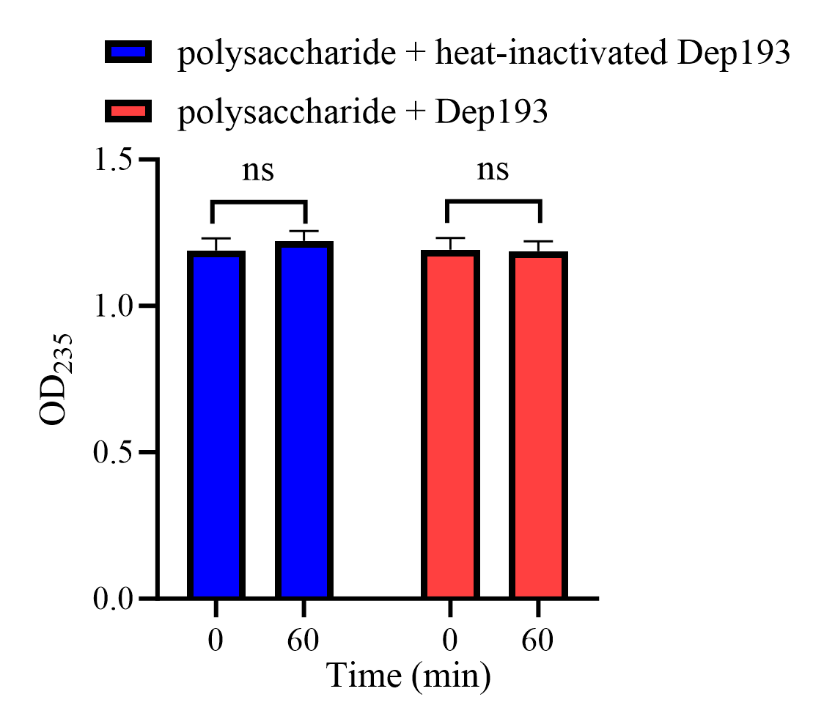


**Fig. S5 Absorbance at 235 nm of the reaction mixture, indicative potential of C4–C5 unsaturated bond intermediate formation.** ns, not significant.


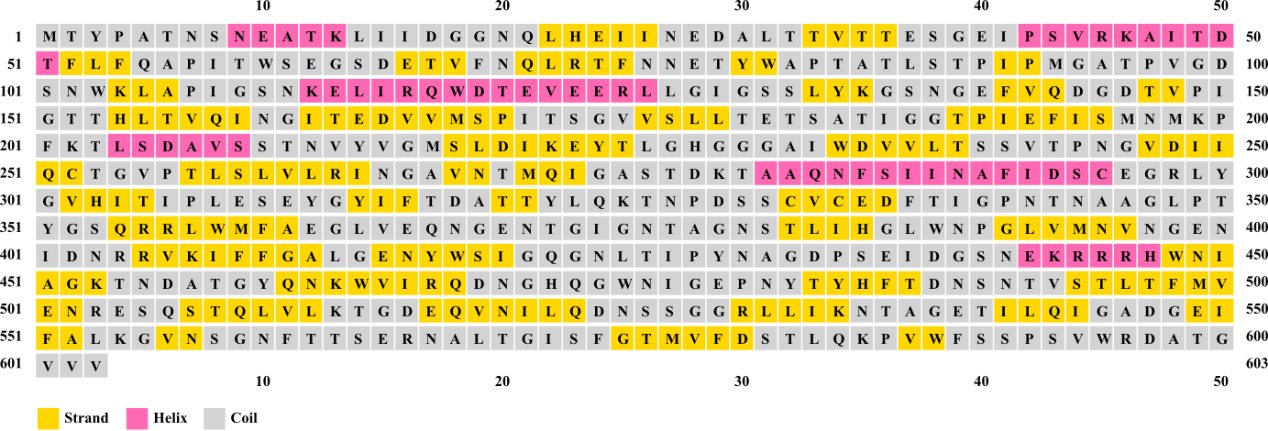


**Fig. S6** **Predicted secondary structure of Dep193.** Secondary structure elements are color-coded: β-strands (yellow), α-helix (pink), coils (gray).


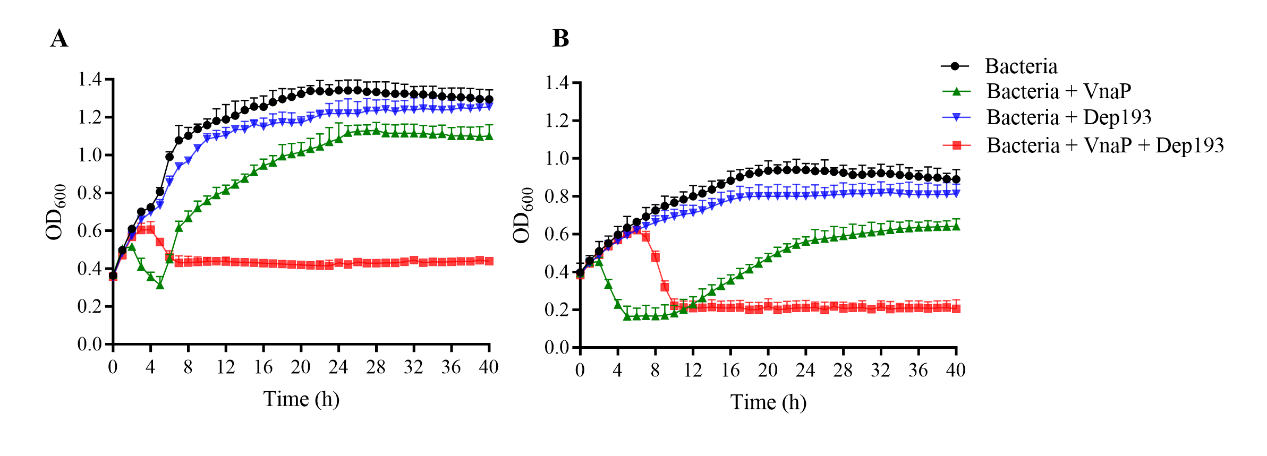


**Fig. S7 Growth curves of** ***V. atypicus* BR18 (A) and** ***V. diabolicus* YN5 (B) under treatment with phage VnaP, Dep193, and their combination.** Data are shown as mean ± SD from three independent experiments.


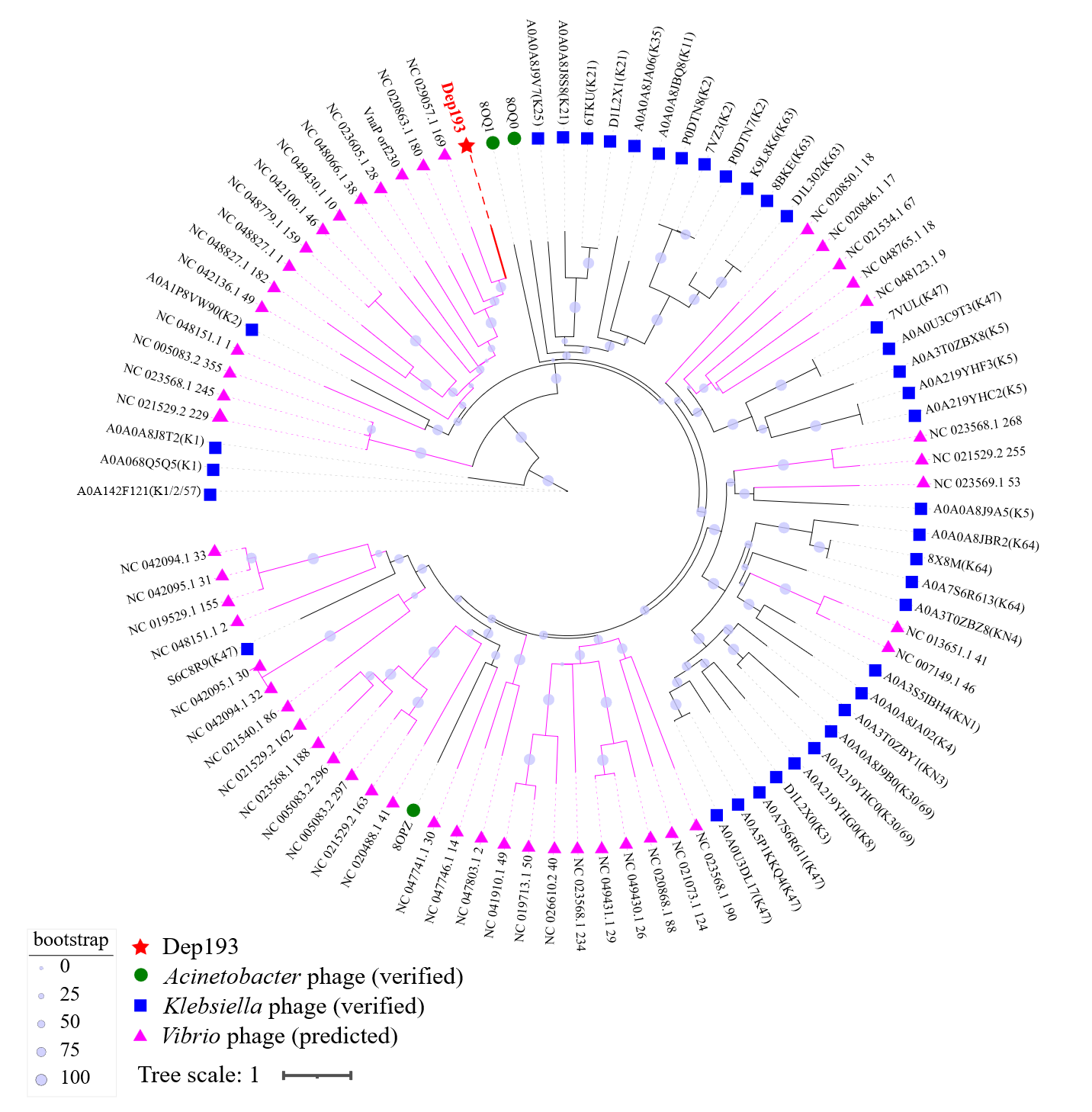


**Fig. S8** **Phylogenetic analysis of Dep193 and related phage depolymerases.** Dep193 (red star) is shown with verified depolymerases from *Acinetobacter* phages (green circles), *Klebsiella* phages (blue squares), and predicted *Vibrio* phage depolymerases (pink triangles). Bootstrap values are indicated by circle size and shading.


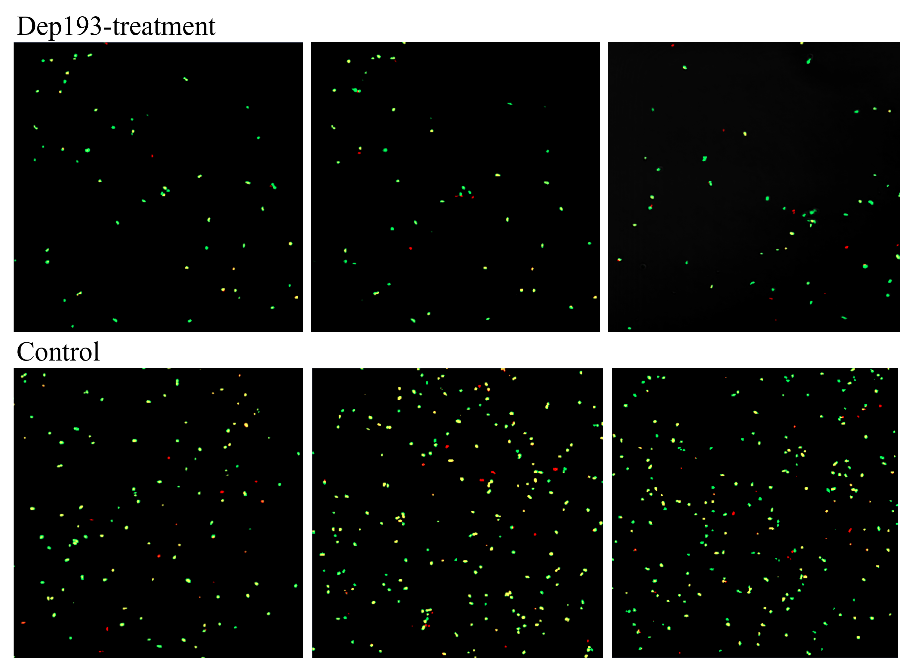


**Fig. S9 Confocal laser scanning microscopy assessment of membrane integrity.** Representative images of *V. natriegens* AbY-1805 cells treated with Dep193 (top row) and PBS control (bottom row). Cells were stained with LIVE/DEAD BacLight kit containing SYTO 9 (Green, live cells) and propidium iodide (Red, dead cells).

**
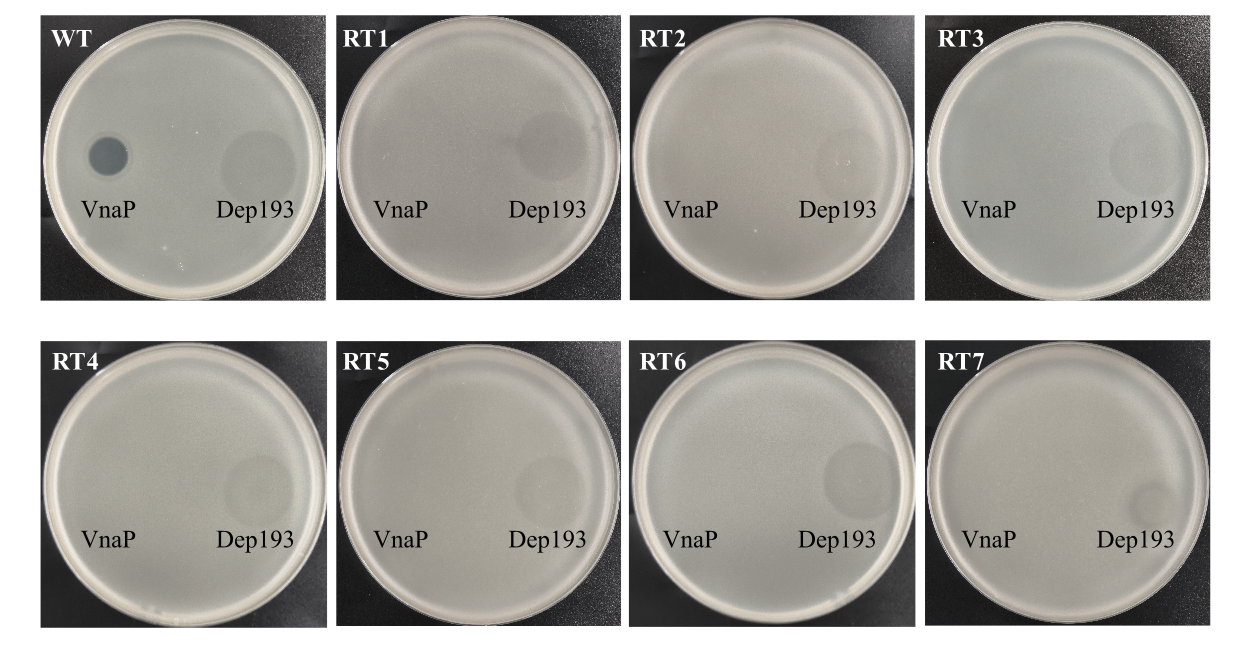
**

**Fig. S10 Antibacterial activity of Dep193 against wild-type (WT) and phage-resistant (RT1****–7) strains determined by spot assay.**

**Tables**

**Table S1 Comparative genomic analysis of phage VnaP and its closest related phages.**

| Hits sequence | Query Coverage | E-value | Percent Identity | Sequence Length | Accession |
| --- | --- | --- | --- | --- | --- |
| *Vibrio* phage VPMCC14 | 25% | 0 | 94.53 | 134472 | ON922990.1 |
| *Vibrio* phage vB_VpaM_R16F | 28% | 0 | 92.4 | 139011 | OP793884.1 |
| *Vibrio* phage vB_VibM_10AMN | 25% | 0 | 93.4 | 121779 | OR472329.1 |
| *Vibrio* phage vB_VnaS-L3 | 1% | 0 | 92.06 | 39988 | ON714422.1 |
| *Vibrio* phage qdvp001 | 23% | 0 | 92.73 | 134742 | NC_029057.1 |
| *Vibrio* phage P018-4 | 19% | 0 | 90.69 | 126958 | PP934186.1 |
| *Vibrio* phage PWH3a-P1 | 13% | 0 | 76.59 | 129155 | NC_020863.1 |
| *Vibrio* phage S4-7 | 3% | 0 | 76.22 | 129756 | KX507046.1 |

**Table S2 Genome annotation of phage VnaP**

| Gene No. | Strand | Start | Stop | Description |
| --- | --- | --- | --- | --- |
| VnaP_001 | + | 240 | 2417 | ATP-dependent Clp protease ATP-binding subunit |
| VnaP_002 | - | 2471 | 3322 | hypothetical protein |
| VnaP_003 | - | 3322 | 3930 | putative head completion adaptor |
| VnaP_004 | + | 3961 | 4266 | hypothetical protein |
| VnaP_005 | - | 4263 | 4763 | hypothetical protein |
| VnaP_006 | - | 4833 | 5858 | major capsid protein E |
| VnaP_007 | - | 5881 | 6267 | putative head stabilization/decoration protein |
| VnaP_008 | - | 6288 | 7715 | SohB, S49 family peptidase |
| VnaP_009 | - | 7854 | 9377 | portal protein |
| VnaP_010 | + | 9661 | 10242 | calcineurin-like phosphoesterase |
| VnaP_011 | + | 10251 | 11138 | putative RNA ligase 1/tail attachment protein |
| VnaP_012 | + | 11128 | 11544 | ATPase |
| VnaP_013 | - | 11655 | 11987 | terminase large subunit |
| VnaP_014 | - | 12238 | 12906 | terminase large subunit |
| VnaP_015 | - | 13157 | 13570 | terminase large subunit |
| VnaP_016 | - | 13803 | 13910 | terminase large subunit |
| VnaP_017 | - | 13910 | 15091 | GIY-YIG catalytic domain |
| VnaP_018 | - | 15200 | 15547 | hypothetical protein |
| VnaP_019 | - | 15540 | 15860 | hypothetical protein |
| VnaP_020 | - | 15857 | 16165 | hypothetical protein |
| VnaP_021 | + | 16274 | 16621 | hypothetical protein |
| VnaP_022 | + | 16621 | 16848 | putative type II toxin-antitoxin system HicB family antitoxin |
| VnaP_023 | - | 17042 | 17401 | hypothetical protein |
| VnaP_024 | - | 17401 | 17865 | TMhelix containing protein |
| VnaP_025 | - | 18791 | 18937 | hypothetical protein |
| VnaP_026 | - | 19008 | 19274 | hypothetical protein |
| VnaP_027 | - | 19274 | 19513 | hypothetical protein |
| VnaP_028 | - | 19515 | 19757 | hypothetical protein |
| VnaP_029 | - | 19761 | 19934 | hypothetical protein |
| VnaP_030 | - | 19934 | 20521 | putative DprA-like protein |
| VnaP_031 | - | 20530 | 21168 | DNA-binding domain protein |
| VnaP_032 | - | 21161 | 22549 | helicase |
| VnaP_033 | - | 22546 | 22728 | hypothetical protein |
| VnaP_034 | - | 22725 | 22922 | hypothetical protein |
| VnaP_035 | - | 22927 | 23271 | TMhelix containing protein |
| VnaP_036 | - | 23268 | 23507 | hypothetical protein |
| VnaP_037 | - | 23500 | 23889 | hypothetical protein |
| VnaP_038 | - | 23892 | 24083 | hypothetical protein |
| VnaP_039 | - | 24095 | 24397 | hypothetical protein |
| VnaP_040 | - | 24397 | 24594 | hypothetical protein |
| VnaP_041 | - | 24596 | 24829 | hypothetical protein |
| VnaP_042 | + | 25630 | 26208 | hypothetical protein |
| VnaP_043 | + | 26210 | 26755 | peptidase HslV family |
| VnaP_044 | + | 26752 | 26928 | hypothetical protein |
| VnaP_045 | + | 26925 | 27167 | hypothetical protein |
| VnaP_046 | + | 27169 | 27426 | hypothetical protein |
| VnaP_047 | + | 27423 | 27665 | hypothetical protein |
| VnaP_048 | + | 27667 | 27942 | hypothetical protein |
| VnaP_049 | + | 27942 | 28577 | deoxynucleoside monophosphate kinase |
| VnaP_050 | + | 28564 | 29160 | putative 5'(3')-deoxyribonucleotidase |
| VnaP_051 | + | 29162 | 29863 | nucleotide pyrophosphohydrolase |
| VnaP_052 | + | 29875 | 30195 | exonuclease |
| VnaP_053 | + | 30205 | 31080 | RdgC recombination associated protein, exonuclease |
| VnaP_054 | + | 31080 | 31187 | hypothetical protein |
| VnaP_055 | + | 31188 | 31322 | hypothetical protein |
| VnaP_056 | + | 31408 | 31572 | hypothetical protein |
| VnaP_057 | + | 31565 | 32587 | HNH endonuclease |
| VnaP_058 | + | 32584 | 33924 | exonuclease activity |
| VnaP_059 | + | 33927 | 34112 | putative type II toxin-antitoxin system HicB family antitoxin |
| VnaP_060 | + | 34113 | 34322 | hypothetical protein |
| VnaP_061 | + | 34335 | 34601 | hypothetical protein |
| VnaP_062 | + | 34607 | 34870 | hypothetical protein |
| VnaP_063 | + | 34873 | 35085 | hypothetical protein |
| VnaP_064 | + | 35085 | 35294 | hypothetical protein |
| VnaP_065 | + | 35295 | 35837 | HNH endonuclease |
| VnaP_066 | + | 35824 | 36027 | hypothetical protein |
| VnaP_067 | + | 36104 | 36259 | hypothetical protein |
| VnaP_068 | + | 36256 | 36819 | endolysin |
| VnaP_069 | + | 36806 | 37018 | hypothetical protein |
| VnaP_070 | + | 37029 | 37241 | hypothetical protein |
| VnaP_071 | + | 37241 | 37537 | hypothetical protein |
| VnaP_072 | + | 37549 | 38841 | metallo-dependent phosphatase-like protein |
| VnaP_073 | + | 38843 | 39019 | hypothetical protein |
| VnaP_074 | + | 39016 | 39471 | nucleotide reductase |
| VnaP_075 | + | 39483 | 39611 | hypothetical protein |
| VnaP_076 | + | 39623 | 40561 | putative thymidylate synthase |
| VnaP_077 | + | 40608 | 40748 | hypothetical protein |
| VnaP_078 | + | 40745 | 41581 | DNA methyltransferase |
| VnaP_079 | + | 41591 | 41950 | hypothetical protein |
| VnaP_080 | + | 42078 | 42611 | hypothetical protein |
| VnaP_081 | + | 42629 | 45880 | putative ribonucleoside diphosphate reductase subunit alpha RnR alpha subunit |
| VnaP_082 | + | 45931 | 47307 | putative ribonucleoside-diphosphate reductase RnR beta subunit |
| VnaP_083 | + | 47368 | 48144 | hypothetical protein |
| VnaP_084 | + | 48203 | 48310 | hypothetical protein |
| VnaP_085 | + | 48332 | 48562 | hypothetical protein |
| VnaP_086 | + | 48559 | 49269 | baseplate hub subunit and tail lysozyme |
| VnaP_087 | + | 49303 | 50088 | putative phosphate starvation-inducible protein PhoH |
| VnaP_088 | + | 50088 | 50702 | HNH endonuclease |
| VnaP_089 | + | 50768 | 50965 | hypothetical protein |
| VnaP_090 | + | 50965 | 51432 | hypothetical protein |
| VnaP_091 | + | 51442 | 52275 | peptidase HslV family |
| VnaP_092 | + | 52329 | 52586 | hypothetical protein |
| VnaP_093 | + | 52586 | 52717 | hypothetical protein |
| VnaP_094 | + | 52777 | 53040 | hypothetical protein |
| VnaP_095 | + | 53043 | 54287 | DNA ligase |
| VnaP_096 | + | 54318 | 54440 | hypothetical protein |
| VnaP_097 | + | 54464 | 54691 | hypothetical protein |
| VnaP_098 | + | 54688 | 54918 | hypothetical protein |
| VnaP_099 | + | 54928 | 55119 | hypothetical protein |
| VnaP_100 | + | 55152 | 55844 | transferase activity |
| VnaP_101 | + | 55890 | 56102 | hypothetical protein |
| VnaP_102 | + | 56099 | 56299 | hypothetical protein |
| VnaP_103 | + | 56310 | 56906 | ATP-dependent Clp protease proteolytic subunit |
| VnaP_104 | + | 56917 | 57081 | hypothetical protein |
| VnaP_105 | + | 57081 | 57299 | hypothetical protein |
| VnaP_106 | + | 57296 | 57742 | phosphatase |
| VnaP_107 | + | 57756 | 57929 | hypothetical protein |
| VnaP_108 | + | 57913 | 58095 | hypothetical protein |
| VnaP_109 | + | 58095 | 58487 | hypothetical protein |
| VnaP_110 | + | 58494 | 58898 | lipocalin family protein |
| VnaP_111 | + | 58886 | 59170 | TMhelix containing protein |
| VnaP_112 | + | 59180 | 59572 | hypothetical protein |
| VnaP_113 | + | 59588 | 59812 | hypothetical protein |
| VnaP_114 | + | 59814 | 60227 | hypothetical protein |
| VnaP_115 | + | 61877 | 62086 | hypothetical protein |
| VnaP_116 | + | 62121 | 62588 | winged helix-turn-helix DNA-binding domain protein |
| VnaP_117 | - | 62621 | 62776 | hypothetical protein |
| VnaP_118 | - | 62886 | 63440 | hypothetical protein |
| VnaP_119 | - | 63542 | 63934 | TMhelix containing protein |
| VnaP_120 | - | 64037 | 64189 | hypothetical protein |
| VnaP_121 | - | 64272 | 64457 | hypothetical protein |
| VnaP_122 | - | 64454 | 64639 | hypothetical protein |
| VnaP_123 | - | 64642 | 64932 | hypothetical protein |
| VnaP_124 | - | 64938 | 65309 | hypothetical protein |
| VnaP_125 | - | 65414 | 65617 | hypothetical protein |
| VnaP_126 | - | 65716 | 66015 | hypothetical protein |
| VnaP_127 | - | 66018 | 66161 | hypothetical protein |
| VnaP_128 | - | 66455 | 66607 | hypothetical protein |
| VnaP_129 | - | 66621 | 66944 | hypothetical protein |
| VnaP_130 | - | 66947 | 67471 | hypothetical protein |
| VnaP_131 | - | 67598 | 67990 | hypothetical protein |
| VnaP_132 | - | 68071 | 68442 | hypothetical protein |
| VnaP_133 | - | 68704 | 69186 | Pyrimidine dimer DNA glycosylase |
| VnaP_134 | - | 69428 | 69685 | hypothetical protein |
| VnaP_135 | - | 69693 | 69935 | hypothetical protein |
| VnaP_136 | - | 69950 | 70300 | hypothetical protein |
| VnaP_137 | - | 70393 | 70725 | hypothetical protein |
| VnaP_138 | - | 70738 | 70893 | hypothetical protein |
| VnaP_139 | + | 71120 | 71284 | hypothetical protein |
| VnaP_140 | - | 72035 | 72487 | hypothetical protein |
| VnaP_141 | - | 72606 | 72887 | hypothetical protein |
| VnaP_142 | - | 72884 | 73267 | hypothetical protein |
| VnaP_143 | - | 73236 | 73565 | hypothetical protein |
| VnaP_144 | - | 73566 | 74066 | hypothetical protein |
| VnaP_145 | - | 74069 | 74170 | hypothetical protein |
| VnaP_146 | - | 74253 | 74348 | DNA methylase |
| VnaP_147 | - | 74380 | 75012 | HNH endonuclease |
| VnaP_148 | - | 75075 | 75197 | DNA methylase |
| VnaP_149 | - | 75209 | 76882 | hypothetical protein |
| VnaP_150 | - | 77089 | 77745 | HNH endonuclease |
| VnaP_151 | - | 77742 | 78347 | hypothetical protein |
| VnaP_152 | - | 78349 | 78594 | hypothetical protein |
| VnaP_153 | - | 78598 | 79020 | hypothetical protein |
| VnaP_154 | - | 79017 | 79532 | coil containing protein |
| VnaP_155 | - | 79532 | 80224 | colanic acid degradation |
| VnaP_156 | - | 80224 | 80424 | hypothetical protein |
| VnaP_157 | - | 80427 | 80684 | hypothetical protein |
| VnaP_158 | - | 80712 | 81050 | hypothetical protein |
| VnaP_159 | - | 81146 | 81439 | hypothetical protein |
| VnaP_160 | - | 81505 | 82608 | hypothetical protein |
| VnaP_161 | - | 82619 | 83347 | serine recombinase |
| VnaP_162 | + | 83421 | 83624 | hypothetical protein |
| VnaP_163 | + | 83621 | 84628 | endonuclease |
| VnaP_164 | - | 84698 | 85216 | hypothetical protein |
| VnaP_165 | - | 85209 | 85601 | hypothetical protein |
| VnaP_166 | - | 85611 | 85772 | hypothetical protein |
| VnaP_167 | - | 85769 | 85924 | hypothetical protein |
| VnaP_168 | - | 85927 | 86199 | hypothetical protein |
| VnaP_169 | - | 86202 | 86567 | hypothetical protein |
| VnaP_170 | + | 86732 | 87523 | HNH endonuclease |
| VnaP_171 | + | 87526 | 87774 | hypothetical protein |
| VnaP_172 | + | 87767 | 88015 | hypothetical protein |
| VnaP_173 | + | 88012 | 88221 | hypothetical protein |
| VnaP_174 | + | 88218 | 88394 | hypothetical protein |
| VnaP_175 | + | 88391 | 88708 | hypothetical protein |
| VnaP_176 | + | 88701 | 88886 | hypothetical protein |
| VnaP_177 | + | 88883 | 89080 | hypothetical protein |
| VnaP_178 | + | 89111 | 89332 | hypothetical protein |
| VnaP_179 | + | 89409 | 89795 | RNase HI |
| VnaP_180 | + | 89786 | 91696 | DNA primase/helicase |
| VnaP_181 | + | 91708 | 93678 | putative DNA polymerase II |
| VnaP_182 | + | 93928 | 94329 | putative DNA polymerase III |
| VnaP_183 | + | 94653 | 94922 | putative DNA polymerase IV |
| VnaP_184 | + | 94977 | 96047 | putative DNA polymerase/3'-5' exonuclease domain V |
| VnaP_185 | + | 96040 | 96237 | hypothetical protein |
| VnaP_186 | + | 96336 | 96611 | hypothetical protein |
| VnaP_187 | + | 96614 | 97195 | catalase-like domain protein |
| VnaP_188 | + | 97258 | 97488 | hypothetical protein |
| VnaP_189 | + | 97490 | 97819 | hypothetical protein |
| VnaP_190 | + | 97821 | 98045 | hypothetical protein |
| VnaP_191 | + | 98038 | 98277 | hypothetical protein |
| VnaP_192 | + | 98483 | 99664 | hypothetical protein |
| VnaP_193 | - | 99745 | 101556 | tail fiber protein |
| VnaP_194 | - | 101566 | 102030 | hypothetical protein |
| VnaP_195 | - | 102040 | 102435 | hypothetical protein |
| VnaP_196 | - | 102461 | 103216 | putative tail fiber protein |
| VnaP_197 | - | 103217 | 103846 | baseplate protein |
| VnaP_198 | - | 103848 | 105326 | putative baseplate component |
| VnaP_199 | - | 105399 | 106067 | baseplate wedge protein |
| VnaP_200 | - | 106067 | 106882 | putative baseplate |
| VnaP_201 | - | 106882 | 107829 | baseplate hub |
| VnaP_202 | - | 107839 | 108216 | virion structural protein |
| VnaP_203 | - | 108219 | 108821 | phage baseplate protein |
| VnaP_204 | - | 108903 | 110183 | endonuclease |
| VnaP_205 | - | 110279 | 112321 | putative tape measure protein |
| VnaP_206 | - | 112314 | 113558 | putative homing endonuclease |
| VnaP_207 | - | 113623 | 113784 | hypothetical protein |
| VnaP_208 | - | 113844 | 114434 | tail assembly chaperone |
| VnaP_209 | - | 114508 | 114978 | putative structural protein |
| VnaP_210 | - | 114991 | 116406 | tail sheath |
| VnaP_211 | - | 116571 | 117233 | hypothetical protein |
| VnaP_212 | - | 117221 | 117745 | minor head protein |
| VnaP_213 | - | 117745 | 118227 | tail completion or Neck1 protein |
| VnaP_214 | - | 118232 | 118453 | hypothetical protein |
| VnaP_215 | + | 118581 | 119219 | putative serine protease |
| VnaP_216 | + | 119230 | 119550 | hypothetical protein |
| VnaP_217 | + | 119613 | 119864 | hypothetical protein |
| VnaP_218 | + | 119866 | 120129 | hypothetical protein |
| VnaP_219 | + | 120129 | 120542 | antimicrobial peptide resistance and lipid A acylation protein PagP |
| VnaP_220 | + | 120645 | 120914 | hypothetical protein |
| VnaP_221 | + | 121121 | 122401 | hypothetical protein |
| VnaP_222 | + | 122412 | 122675 | hydroxymethylglutaryl-CoA reductase (NADPH) activity |
| VnaP_223 | + | 122798 | 122980 | hypothetical protein |
| VnaP_224 | + | 122980 | 123186 | hypothetical protein |
| VnaP_225 | + | 123189 | 123770 | Mom-like DNA modification protein |
| VnaP_226 | + | 123781 | 124002 | tRNA pseudouridine synthase D |
| VnaP_227 | + | 124142 | 126280 | anaerobic ribonucleoside-triphosphate reductase |
| VnaP_228 | + | 126277 | 126735 | anaerobic ribonucleoside triphosphate reductase activating protein |
| VnaP_229 | - | 126753 | 126881 | hypothetical protein |
| VnaP_230 | + | 127084 | 128775 | colanic acid degradation |
| VnaP_231 | + | 128763 | 129242 | hypothetical protein |

**Table S3 BLASTp-based sequence similarity analysis of depolymerase Dep193.**

| Database | Description | Origin | Query Cover | E-value | Percent Identity | Accession |
| --- | --- | --- | --- | --- | --- | --- |
| nr | hypothetical protein | *Marinilactibacillus psychrotolerans* | 66% | 5.00E-105 | 45.81% | WP_220726828.1 |
| nr | hypothetical protein | *Vibrio* phage PWH3a-P1 | 52% | 6.00E-70 | 50.00% | YP_007676032.1 |
| nr | tail fiber protein | *Vibrio* phage IBT_VpB-34 | 51% | 5.00E-62 | 44.84% | WQZ33736.1 |

**Table S4** **Foldseek-based structural homology analysis of depolymerase Dep193.**

| Database | Description | Origin | TM-score | RMSD | Accession |
| --- | --- | --- | --- | --- | --- |
| PDB | tailspike protein gp49 | *Acinetobacter* phage Fri1 | 0.40 | 6.76 | 6C72 |
| PDB | Tailspike protin orf212 | *Kuttervirus* CBA120 | 0.34 | 7.53 | 6NW9 |
| PDB | Tailspike protin orf213 | *Kuttervirus* CBA120 | 0.34 | 7.87 | 5W6H |
| PDB | Tailspike protein | *Acinetobacter* phage AM24 | 0.35 | 7.00 | 5W5P |
| PDB | hypothetical protein | *Kuttervirus* CBA120 | 0.30 | 7.04 | 6W4Q |
| AFDB-SWISSPROT | Tail fiber protein | *Haemophilus influenzae* Rd KW20 | 0.34 | 7.48 | P44242 |

**Table S5 Experimentally validated phage depolymerases from public databases used in this study**

| Accession | DePP score | Origin | Database |
| --- | --- | --- | --- |
| A0A219YHC2 | 0.96 | *Klebsiella* phage | UniProt |
| A0A219YHF3 | 0.96 | *Klebsiella* phage | UniProt |
| D1L2X0 | 0.95 | *Klebsiella* phage | UniProt |
| A0A7S6R611 | 0.95 | *Klebsiella* phage | UniProt |
| A0A7S6R613 | 0.95 | *Klebsiella* phage | UniProt |
| P0DTN7 | 0.95 | *Klebsiella* phage | UniProt |
| A0A3S5IBH4 | 0.95 | *Klebsiella* phage | UniProt |
| P0DTN8 | 0.95 | *Klebsiella* phage | UniProt |
| A0A0A8J9A5 | 0.95 | *Klebsiella* phage | UniProt |
| A0A5P1KKQ4 | 0.95 | *Klebsiella* phage | UniProt |
| S6C8R9 | 0.94 | *Klebsiella* phage | UniProt |
| A0A0A8JBQ8 | 0.94 | *Klebsiella* phage | UniProt |
| K9L8K6 | 0.94 | *Klebsiella* phage | UniProt |
| A0A0A8J8T2 | 0.94 | *Klebsiella* phage | UniProt |
| A0A3T0ZBY1 | 0.94 | *Klebsiella* phage | UniProt |
| A0A0A8JA06 | 0.94 | *Klebsiella* phage | UniProt |
| A0A0A8J8S8 | 0.94 | *Klebsiella* phage | UniProt |
| A0A0A8JBR2 | 0.94 | *Klebsiella* phage | UniProt |
| D1L302 | 0.93 | *Klebsiella* phage | UniProt |
| A0A0A8JA02 | 0.93 | *Klebsiella* phage | UniProt |
| A0A0A8J9V7 | 0.93 | *Klebsiella* phage | UniProt |
| A0A0U3C9T3 | 0.93 | *Klebsiella* phage | UniProt |
| A0A068Q5Q5 | 0.93 | *Klebsiella* phage | UniProt |
| A0A219YHC0 | 0.93 | *Klebsiella* phage | UniProt |
| A0A3T0ZBX8 | 0.93 | *Klebsiella* phage | UniProt |
| A0A1P8VW90 | 0.93 | *Klebsiella* phage | UniProt |
| D1L2X1 | 0.92 | *Klebsiella* phage | UniProt |
| A0A0U3DL17 | 0.92 | *Klebsiella* phage | UniProt |
| A0A219YHG0 | 0.92 | *Klebsiella* phage | UniProt |
| A0A142F121 | 0.92 | *Klebsiella* phage | UniProt |
| A0A0A8J9B0 | 0.92 | *Klebsiella* phage | UniProt |
| A0A3T0ZBZ8 | 0.91 | *Klebsiella* phage | UniProt |
| 8X8M | 0.96 | *Klebsiella* phage | PDB |
| 8BKE | 0.93 | *Klebsiella* phage | PDB |
| 7VZ3 | 0.95 | *Klebsiella* phage | PDB |
| 7VUL | 0.95 | *Klebsiella* phage | PDB |
| 6TKU | 0.92 | *Klebsiella* phage | PDB |
| 8OQ1 | 0.92 | *Acinetobacter* phage | PDB |
| 8OQ0 | 0.94 | *Acinetobacter* phage | PDB |
| 8OPZ | 0.92 | *Acinetobacter* phage | PDB |

**References**

1. **Li C, Wang Z, Zhao J, Wang L, Xie G, Huang J, Zhang Y**. 2021. A novel *Vibrio* phage vB_VcaS_HC containing lysogeny-related gene has strong lytic ability against pathogenic bacteria. Virol Sin 36:281–290.
2. **Liu Y, Mi Z, Mi L, Huang Y, Li P, Liu H, Yuan X, Niu W, Jiang N, Bai C, Gao Z**. 2019. Identification and characterization of capsule depolymerase Dpo48 from Acinetobacter baumannii phage IME200. PeerJ 7:e6173.
3. **Li C, Shi T, Sun Y, Zhang Y.** 2022. A Novel Method to Create Efficient Phage Cocktails via Use of Phage-Resistant Bacteria. *Appl Environ Microbiol* 88:e02323–21.
4. **Yadav S, Yadav PK, Yadav D, Yadav KDS.** 2009. Pectin lyase: A review. *Process Biochem* 44:1–10.
5. **Yang P, Shan B, Hu X, Xue L, Song G, He P, Yang X.** 2025. Identification of a novel phage depolymerase against ST11 K64 carbapenem-resistant Klebsiella pneumoniae and its therapeutic potential. *J Bacteriol* 207:e00387–24.
